# Immunotherapy using IgE or CAR T cells for cancers expressing the tumor antigen SLC3A2

**DOI:** 10.1136/jitc-2020-002140

**Published:** 2021-06-10

**Authors:** Giulia Pellizzari, Olivier Martinez, Silvia Crescioli, Robert Page, Ashley Di Meo, Silvia Mele, Giulia Chiaruttini, Jan Hoinka, Ihor Batruch, Ioannis Prassas, Melanie Grandits, Jacobo López-Abente, Eva Bugallo-Blanco, Malcolm Ward, Heather J Bax, Elise French, Anthony Cheung, Sara Lombardi, Mariangela Figini, Katie E Lacy, Eleftherios P Diamandis, Debra H Josephs, James Spicer, Sophie Papa, Sophia N Karagiannis

**Affiliations:** 1 St John's Institute of Dermatology, School of Basic and Medical Biosciences, King's College London, London, England, UK; 2 Immunoengineering Group, King's College London, London, England, UK; 3 Lunenfeld-Tanenbaum Research Institute, Mount Sinai Hospital, Toronto, Ontario, Canada; 4 Computational Biology Branch, National Center for Biotechnology Information, National Library of Medicine, National Institutes of Health, Bethesda, Maryland, USA; 5 Department of Pathology and Laboratory Medicine, Mount Sinai Hospital, Toronto, Ontario, Canada; 6 Aulesa Biosciences Ltd, Shefford, England, UK; 7 Breast Cancer Now Research Unit, School of Cancer and Pharmaceutical Sciences, King's College London, London, England, UK; 8 School of Cancer and Pharmaceutical Sciences, King's College London, London, England, UK; 9 Biomarker Unit, Dipartimento di Ricerca Applicata e Sviluppo Tecnologico (DRAST), Fondazione IRCCS Istituto Nazionale dei Tumori, Milan, Italy; 10 Department of Laboratory Medicine and Pathobiology, University of Toronto, Toronto, Ontario, Canada; 11 Department of Clinical Biochemistry, University Health Network, Toronto, Ontario, Canada; 12 Department of Medical Oncology, Guy's and St Thomas' NHS Foundation Trust, London, England, UK

**Keywords:** immunotherapy, receptors, chimeric antigen, cytokines, antibody specificity, immunoassay

## Abstract

**Background:**

Cancer immunotherapy with monoclonal antibodies and chimeric antigen receptor (CAR) T cell therapies can benefit from selection of new targets with high levels of tumor specificity and from early assessments of efficacy and safety to derisk potential therapies.

**Methods:**

Employing mass spectrometry, bioinformatics, immuno-mass spectrometry and CRISPR/Cas9 we identified the target of the tumor-specific SF-25 antibody. We engineered IgE and CAR T cell immunotherapies derived from the SF-25 clone and evaluated potential for cancer therapy.

**Results:**

We identified the target of the SF-25 clone as the tumor-associated antigen SLC3A2, a cell surface protein with key roles in cancer metabolism. We generated IgE monoclonal antibody, and CAR T cell immunotherapies each recognizing SLC3A2. In concordance with preclinical and, more recently, clinical findings with the first-in-class IgE antibody MOv18 (recognizing the tumor-associated antigen Folate Receptor alpha), SF-25 IgE potentiated Fc-mediated effector functions against cancer cells in vitro and restricted human tumor xenograft growth in mice engrafted with human effector cells. The antibody did not trigger basophil activation in cancer patient blood ex vivo, suggesting failure to induce type I hypersensitivity, and supporting safe therapeutic administration. SLC3A2-specific CAR T cells demonstrated cytotoxicity against tumor cells, stimulated interferon-γ and interleukin-2 production in vitro. In vivo SLC3A2-specific CAR T cells significantly increased overall survival and reduced growth of subcutaneous PC3-LN3-luciferase xenografts. No weight loss, manifestations of cytokine release syndrome or graft-versus-host disease, were detected.

**Conclusions:**

These findings identify efficacious and potentially safe tumor-targeting of SLC3A2 with novel immune-activating antibody and genetically modified cell therapies.

## Background

Recent years have seen the successful translation of immunotherapy strategies to the clinic with monoclonal antibodies being at the forefront of efficacious treatments. Similarly, the development of genetically modified cell therapy approaches utilizing synthetic chimeric antigen receptor (CAR) T cell technologies, has resulted in three cluster of differentiation-19 (CD19) and one B-cell maturation antigen targeting product receiving FDA approval for hematological cancers. Suitable tumor associated antigens (TAAs) for antibody and CAR T cell therapies require cell surface expression on cancer cells, and low/restricted distribution in normal tissues. These requirements critically limit the choice of TAAs suitable for monoclonal antibody and CAR T cell therapy development.

In 1988, Wilson *et al.* investigated the antigenic changes correlated with malignant transformation of hepatocytes: their research was focused on the detection of common antigens among tissues derived from the same germ layer that could be associated with transformed cell phenotypes.^1^ Mice were immunized with the hepatocellular carcinoma cell line FOCUS and hybridomas were developed. Subsequent screening identified 18 antibodies reactive against human colon carcinoma cell lines. The murine IgG_1_ SF-25 antibody clone was chosen for further investigation.^1^ SF-25 bound 17 out of 17 human colon adenocarcinoma biopsies, while demonstrating no staining of normal adjacent mucosa. In vivo localization to subcutaneous tumors in nude mice was demonstrated. These data confirmed colon-cancer specificity for SF-25.^2^ Subsequently, various steps to develop the SF-25 clone were undertaken, including engineering of a human/murine IgG_1_ chimeric antibody,^3^ development as a positron emission tomography imaging tracer,^4^ and as an antibody drug conjugate (ADC).^5 6^ The chimeric SF-25 antibody has also been used to improve the targeting and effector function of adoptively transferred lymphokine activated killer cells to cancer cells in a liver metastasis model of colorectal cancer.^7^ These data highlighted the potential of the SF-25 clone for cancer specific drug discovery. However, the target antigen of SF-25 remained elusive, hampering further clinical development.

Monoclonal antibodies represent a well-established platform to combat cancer. Until recently only those of the IgG class have been employed for cancer immunotherapy. Since different antibody classes function through unique Fc-receptors and induce specific immune responses at different anatomical sites, the design of new therapeutics could exploit antibody isotypes other than IgG. IgE antibodies, well known for their pathogenic roles in allergic disease, may offer multiple advantages over those conferred by IgG in treating solid malignancies.^8^ These are based on known aspects of IgE biology, commonly employed in immune protection from parasites and in allergic responses, which may translate to superior efficacy in targeting tumors. Such attributes of the IgE class include: (1) high affinity for IgE Fc epsilon receptors (FcεRs) (2–5 orders of magnitude greater than that of IgGs for FcγRs), (2) expression of FcεRs on a distinct spectrum of tissue-resident and tumor-resident immune effector cells, (3) long tissue residency and retention of IgEs by immune effector cells, (4) lack of an inhibitory Fc receptor and (4) active immune surveillance in Th2-biased environments in tissues such as the skin and gut. The potential efficacy of antitumor IgEs recognizing cancer antigens has been demonstrated in several in vivo and in vitro models. First in class human data for IgE utility in ovarian cancer (NCT02546921) has reported interim promising safety and biological activity.^9^

Translating the success of CD19 targeting CAR T cells into solid tumors is hampered by numerous challenges. Namely, homing to tumor sites, persistence of adoptively transferred cells, development of exhausted phenotypes on adoptive transfer, and on-target off-tumor killing in healthy tissues. The apparent tumor specificity of the SF-25 antibody raised the potential that this antibody, and its elusive target, could be a promising axis for the development of novel strategies for IgE and cell-based immunotherapy.

Here, we undertook to identify the target of SF-25 on human cells by utilizing a bioinformatics and mass spectrometry pipeline. We then designed IgE and CAR T cell approaches to demonstrate the potential for broad cancer immunotherapy development.

## Methods

### Cloning and production of recombinant antibodies

To clone the SF-25 variable region into a human IgG_1_ backbone we performed a four-fragment Polymerase Incomplete Primer Extension (PIPE) PCR: two big fragments F2 and F4 (3000–4000 bp) containing at 5’ the constant regions of the light and heavy chain, respectively; and two small fragments F1 and F3 (300–400 bp) composed by SF-25 Vk and VH, respectively.^10–12^ The PCR reactions contained 0.5 µM of each primer, 25 µL of PhusionTM Flash High-Fidelity PCR Master Mix, 10 ng of template DNA and sterile water up to 50 µL. The cycles for amplification were: 10 s at 98°C, 35 cycles of 1 s at 98°C, 5 s at 62°C, and 10 s at 72°C. PCR products were treated with DpnI, bacteria were transformed with equal amount of the digested PCR products to combine the four fragments and plated in Luria Bertani (LB) agar plates supplemented with 200 µg/mL hygromycin B. Colonies were amplified overnight in LB supplemented with 200 µg/mL hygromycin B and DNA was extracted with a QIAprep spin miniprep kit (Qiagen). Correct assembly of the plasmid was verified by sending the newly generated and extracted plasmid for sequencing (Source Bioscience). The sequencing output was analyzed using FinchTV.

To obtain the pVitro1-SF-25 IgE expression vector, we designed the primers for a three fragment PIPE cloning protocol, amplifying the fragment containing the epsilon heavy chain constant region (PCR1) from an in-house representative pVitro1-IgE vector and the other two fragments (PCR2 and PCR3) from our pVitro1-SF25-IgG_1_. The cycles for PCR1 and 2 were: 10 s at 98°C, 35 cycles of 1 s at 98°C, 5 s at 62°C, and 10 s at 72°C. PCR3 was performed with 10 s at 98°C, 35 cycles of 1 s at 98°C, 5 s at 62°C, and 8 s at 72°C. PCR products were separated on 1% agarose gels to discriminate the multiple PCR products by molecular weight. DNA was purified from the gel using the PureLinkTM Quick Gel Extraction Kit (ThermoFisher). PCR products were treated with DpnI to digest the template DNA. One-Shot TOP10 bacteria were transformed with equal amount of the digested PCR products to combine the four fragments (F1-F2-F3-F4) and generate the pVitro1 SF-25 IgG_1_ vector. The correct assembly of the plasmid was verified via sequencing and analyzed using FinchTV. Antibodies were produced in Sp2/0 (IgE) and Expi293F (IgG) mammalian cells and purified using previously described methods (see online supplementalry materials and methods).^11–13^

10.1136/jitc-2020-002140.supp1 Supplementary data

### SF-25 antigen expression screening

Cell lines were detached with Trypsin-EDTA treatment, counted and 2.5×10^5^ cells were used per tube. 2 mL FACS buffer [Phosphate Buffer Saline (PBS); 5% Foetal Bovine Serum (FBS); 3 mM Ethylenediaminetetraacetic acid (EDTA)] was added to each tube before a 5 min centrifugation at 400rcf at 4°C. Cells were resuspended in 100 µL FACS buffer and incubated for 20 min at 4°C with a range of concentrations from 0 to 50 ng SF-25 IgG_1_ or 0 to 5 µg SF-25 IgE. Cells were washed with 2 mL FACS Buffer and were then incubated with goat antihuman IgG-FITC (Fluorescein isothiocyanate) or antihuman IgE-FITC for 20 min at 4°C. Cells were washed with 2 mL FACS buffer and resuspended in 400 µL FACS buffer for cytometry analysis. Analysis of flow cytometry data was performed by FlowJo (TreeStar Inc) software.

### SF-25 antigen: bioinformatics analysis

SF-25 antibody binding scores were generated from three experimental datasets, two binding datasets generated for this study by Flow Cytometry and one previously published radioligand binding dataset.^2^ Transcriptome RNAseq datasets E-MTAB-2706; E-MTAB-2770 and E-MTAB-3983, including several of the human cancer cell lines present in the binding datasets, were downloaded and filtered to only keep the cell lines for which binding data were available. Further filtering and sorting steps were performed to generate matching tables between one binding dataset and the transcription level files. The matched tables were then analyzed, and Spearman correlation scores were calculated for each individual gene in each binding experiment. Average Spearman scores and their variances were generated across the three different combinations made. Further details on the bioinformatic process design and coding are reported in online supplementalry materials and methods.

### Cytotoxicity/phagocytosis (antibody dependent cell-mediated cytotoxicity/ADCP) assay and Basophil activation test

Antibody dependent cell-mediated cytotoxicity/phagocytosis (ADCC)/ADCP assays were performed according to a previously described method.^14^ The ability of SF-25 IgE to trigger primary human basophil activation was determined using an ex vivo assay in which CD63 expression on the surface of human basophils was used as an early marker of basophil activation, as described previously (see online supplemental materials and methods).^15^

### Cytotoxicity assays with CAR T cells

After 24 hours coculture, the viability of tumor cell monolayers was quantified. T cells were removed from the wells and MTT (3-(4,5-dimethylthiazol-2-yl)−2,5-diphenyltetrazolium bromide, Sigma) was added at 500 µg/mL in 200 µL complete DMEM medium and incubated for 1 hour at 37°C and 5% CO_2_. After removal of the supernatant, formazan crystals were resuspended in 200 µL DMSO. Absorbance was measured at 560 nm using a spectrophotometric plate reader (FluoSTAR Omega) and tumor cell viability percentage was calculated as follows: (absorbance of coculture/absorbance of monolayer alone) x 100. Further details of CAR T in vitro and in vivo assays in online supplemental materials and methods.

## Results

### Engineered SF-25 antibodies with human Fc regions recognize human malignant cell lines

**Figure 1:**
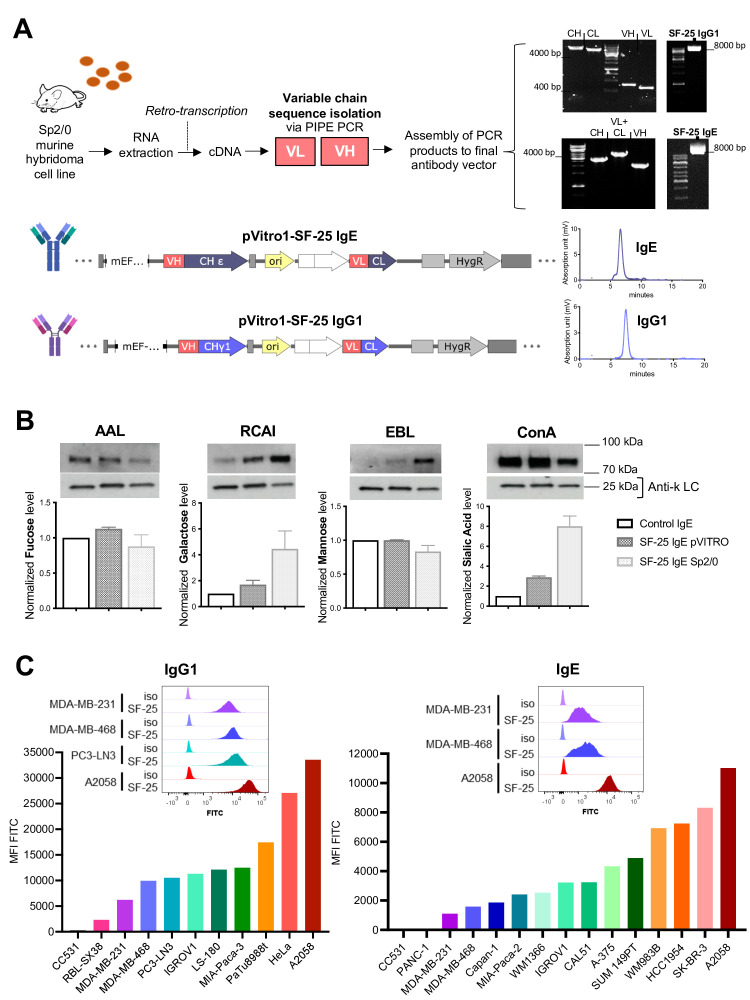
Engineering and testing of SF-25 antibodies with human IgG_1_ or IgE Fc regions. (A) Schematic depicting the steps to obtain the variable regions of the SF-25 antibody and representative images for the visualization of the PCR products on agarose gels, followed by final sequence constructs of pVitro1 expression vectors to produce SF-25 IgE or IgG1 full length chimeric antibodies and the HPLC elution profile of SF-25 IgE and IgG_1_. (B) Lectin blot data reporting the glycosylation profile of SF-25 IgE pVITRO and SF-25 IgE Sp2/0 for fucose, galactose, mannose and sialic acid (detected, respectively, with AAL-biotin (Aleuria aurantia lectin), RCAI-biotin (Ricinus communis agglutinin I lectin), Con-A-biotin (concanavalin a lectin) or EBL (Sambucus nigra lectin)). (C) Flow cytometric data depicting SF-25 IgG_1_ (left panel) and IgE (right panel) binding to different cell lines. Values are reported as mean fluorescence intensities (MFI) detected with antihuman IgG or IgE (respectively) FITC-conjugated secondary antibodies. Representative binding profiles for cell lines targeted in the present study (top panels) (see also online supplemental figure S1). Mouse image used courtesy of MCKIBILLO. HPLC, High performance liquid chromatography.

We cloned the murine variable region sequences of SF-25 into human IgG_1_ and IgE antibody scaffolds (figure 1A) using previously established platforms.^10–12^ High performance liquid chromatography (HPLC) analyses demonstrated high antibody purity and negligible aggregation (<5%) (figure 1B). Production of high-purity intact antibody was demonstrated in different expression systems, culture media, serum content and culture vessel conditions (online supplemental figure S1A, B). Serum-free, serum depleted, or specialist serum-free (ADCF, animal-derived component-free) media all led to high purity antibody with negligible protein aggregates or non-assembled light chain (online supplemental figure S1C, D). Intact antibody can be generated at small and large scale in serum-free conditions demonstrating utility for preclinical process development and clinical testing.

IgE class antibodies are highly glycosylated (12% of molecular weight). We evaluated IgE glycosylation in two SF-25 IgEs produced using mouse (Sp2/0) and human (Expi293F) expression systems.^16 17^ Lectin blot analyses were performed for the most common sugars known to decorate antibodies expressed in mammalian systems: mannose, fucose, galactose and sialic acid. No significant difference in fucose and mannose was observed between murine and human expression systems. Sp2/0-derived SF-25 IgE contained 2.5 times higher galactose and sialic acid content compared with IgE engineered in human Expi293F (figure 1B).

We confirmed the reactivity of each SF-25 chimeric SF-25 IgG_1_ and IgE against a panel of human and non-human tumor cell lines. We showed largely comparable reactivity between IgG and IgE and variable binding across human tumor cell lines, likely reflective of target expression levels. Overall, 18 out of 19 human cancer cell lines from 7 different origins tested were recognized by SF-25 antibodies. Antigen density varied, but with clear population shifts demonstrated for the cell lines tested (figure 1C). No or low binding were detected to the non-human rat colon carcinoma cell line CC531.

Together, these findings confirm the generation of intact, monomeric human Fc IgG_1_ and IgE SF-25, and production of functional IgE in serum free-culture mammalian expression systems suitable for future translation. We confirmed binding of the antibodies to human tumor cells of different malignant origins in agreement with reports of the original SF-25 mouse clone.

### Identification of the target of the SF-25 antibody clone with engineered antibodies

**Figure 2:**
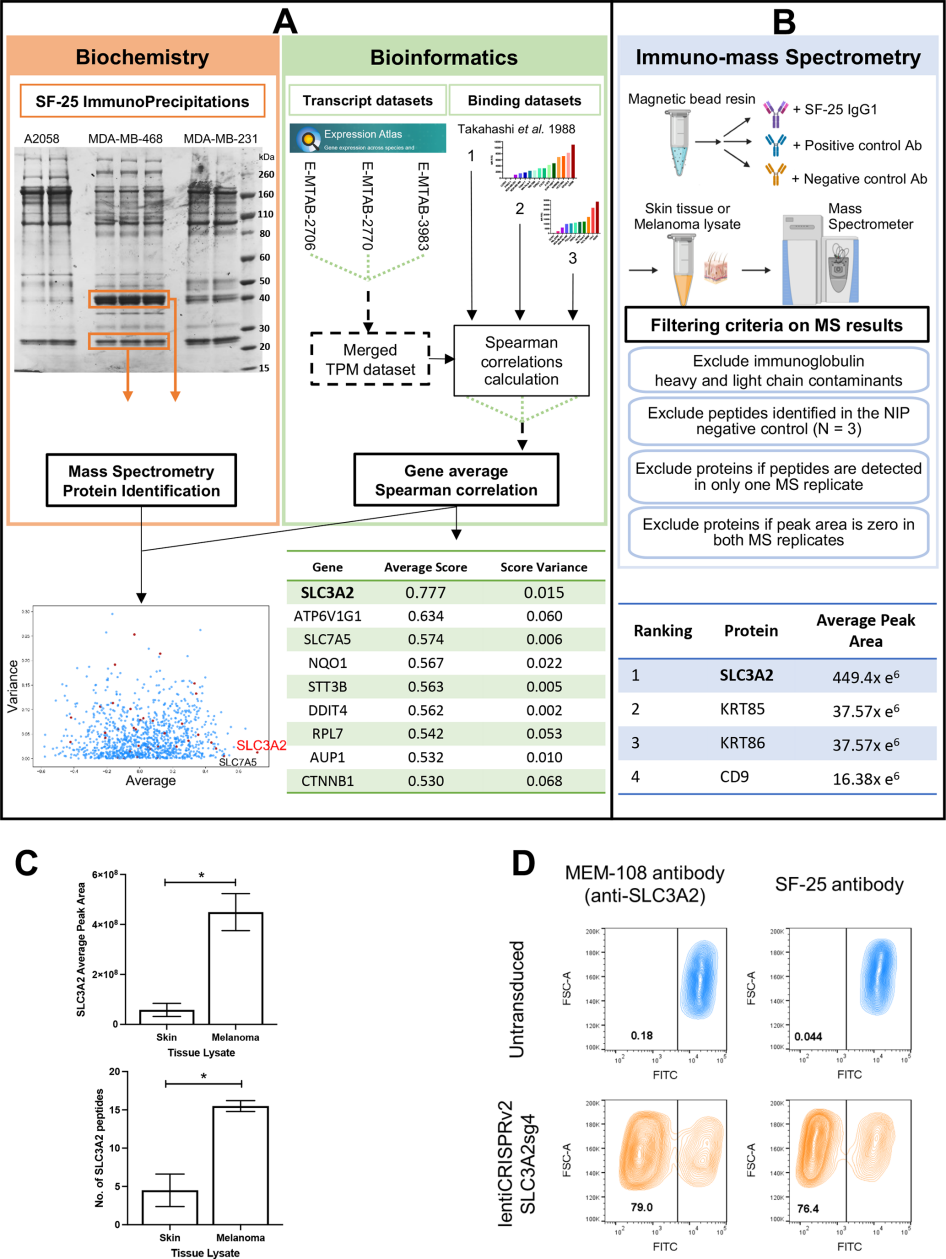
The SF-25 antibody clone recognizes the human CD98 heavy chain (CD98hc) coded for by *SLC3A2*. (A) Immunoprecipitation-mass spectrometry (IP-MS) evaluations identified candidate proteins (left panel). In parallel, transcriptomic and bioinformatics studies identified candidate proteins (right panel). Analyses were matched to identify the target of the SF-25 antibody (bottom panel). (B) Immuno-MS was performed on lysates from human skin (n=3) and human melanoma (n=2) specimens. In two independent experiments, recognition of more than one antigenic peptide from one antigen confirmed antigen reactivity. Binding results were subjected to sequential filtering criteria: first, Ig heavy and light chain contaminants were removed; then peptides that were identified in negative and positive antibody controls were excluded; peptides bound in only one of the two MS injections were removed; and finally, if the peak area of the MS detection peptide profile was zero in both tests. Proteins were ranked based on the average peak area of antibody-bound peptides identified across two independent experiments of melanoma specimens (bottom panel). (C) The average peak area and the cumulative number of SLC3A2 peptides identified via SF-25 IgG_1_ binding to two melanoma and three normal skin specimens by immuno-MS each tested in two injections and across two independent experiments (*p<0.05). (D) The target identity of SF-25 antibody clone was confirmed on SLC3A2 CRISPR KO A2058 cells: the reactivity of the SF-25 clone and a commercially available (MEM-108) to cancer cells was impaired in transduced tumor cells (see also online supplemental figure S2).

Three complementary approaches were employed to identify the target of SF-25. We performed immunoprecipitations with the chimeric SF-25 IgG1 on cell lysates from three different human cancer cell lines with differing SF-25 target expression levels (figure 1C): MDA-MB-231, MDA-MB-468 (breast cancer, BRCA) and A2058 (melanoma) (figure 2A-left panel). No specific protein band emerged by comparing these three samples based on the predicted target abundance. However, bands at 27 and 42 kDa were visible across the three cell lines. They were further analyzed by trypsin digestion and mass spectrometry. Peptides belonging to 138 different proteins were identified (online supplemental table S1). A transcriptomic analysis was performed to compare binding levels on panels of human cancer cell lines against transcript levels for each gene in the same cell lines (figure 2A-right panel). Three sets of binding scores were generated reflecting the binding intensity measured for the SF-25 antibody on each cell line, counts per minute for previous radioligand binding data,^2^ and mean fluorescent intensity (MFI) for the experiments in figure 1C. The relative binding scores for the three binding panels are shown in online supplemental table S2. The binding intensities observed across different cell lines in one binding experiment were compared with transcript levels in aggregate RNA-seq data for the same cell lines. A Spearman correlation score was calculated between SF-25 binding and transcript levels for each gene and average Spearman scores and variance values were calculated from the three comparative studies. The heavy chain of CD98 (CD98hc) encoded by the solute carrier family 3 member 2 (SLC3A2) demonstrated the highest Spearman score (figure 2A-bottom panel). The gene SLC7A5, coding a binding partner of CD98hc, was the second highest hit. Both partners were identified among the candidates immunoprecipitated (figure 2A–bottom panel). SLC3A2 was also identified among 138 different proteins after immunoprecipitation, offering a weak biochemical validation. We, therefore, identified SLC3A2 as the top candidate.

In separate immuno-mass spectrometry experiments, SF-25 IgG_1_ reactivity was tested against human cutaneous and tumor antigens. This immuno-mass spectrometry method can be applied to discover proteome-wide targets of antibodies by using complex protein mixtures from human tissues as sources of candidate proteins.^18^ Tissue lysates were generated from three pooled human skin samples, two human cutaneous metastatic melanoma lesions, and the human ovarian carcinoma cell line IGROV1. In two independent experiments, applying filtering for contaminates and non-specific binding using a non-binding (hapten-specific) antibody (figure 2B), 10 and 7 peptides corresponding to SLC3A2 were identified from the three skin samples. Separately, 18 peptides from two human melanoma lesion samples and 16 peptides from the IGROV1 cell line identified recognition of SLC3A2 by the antibody (figure 2B). The average peak area of binding to melanoma cells and normal skin across two independent experiments was significantly higher in malignant cells for SLC3A2, demonstrating highest expression of the target in tumor samples (figure 2C). In concordance, the identified SLC3A2 peptides (online supplemental figure S2) were demonstrated at significantly higher levels in melanoma vs normal skin (p<0.05, data from three normal skin and two melanoma samples tested twice each in two independent experiments) (figure 2C). Finally, using single guide CRISPR Cas-9 knock out of the SLC3A2 gene in A2058 cells, comparable loss of binding was seen with SF-25 IgG and a commercially available CD98hc specific monoclonal antibody (figure 2D).

These data identify the protein CD98hc, coded for by SLC3A2, as the target of the SF-25 antibody clone both bioinformatically and biochemically.

### The SLC3A2 derived protein CD98hc is a tumor-associated-antigen expressed on a broad range of human tumors with limited normal tissue expression

**Figure 3:**
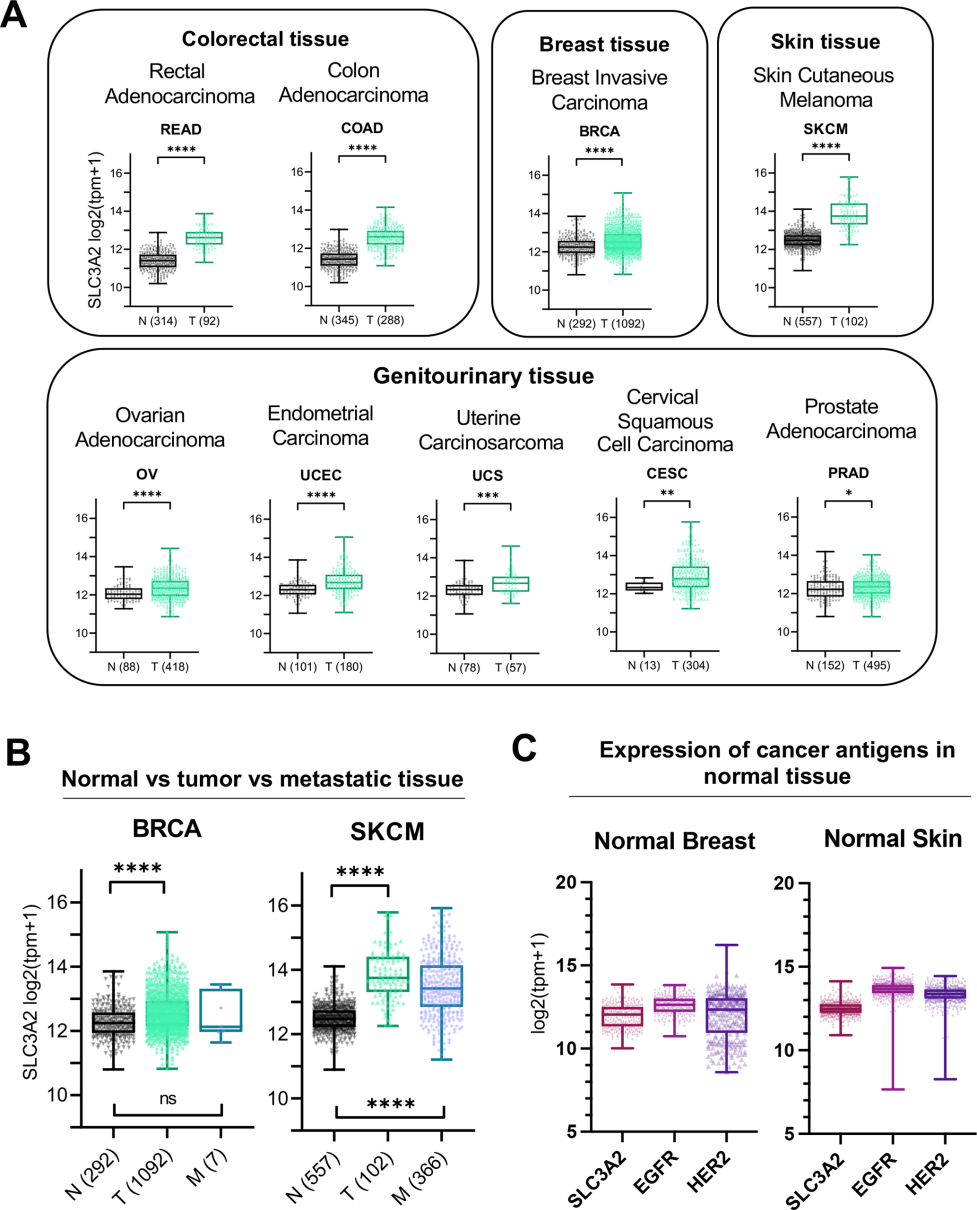
*SLC3A2* gene expression is enhanced in different malignancies compared with equivalent normal tissues. (A) Expression of *SLC3A2* in normal (N) versus tumor (T) tissues of different origins (separated by tissue type; colorectal and colon adenocarcinomas, breast, skin and genitourinary tissues). Tumor types are described in online supplemental materials and methods *—SLC3A2* differential expression study). (B) *SLC3A2* gene expression primary tumor (T) and tumor metastases (M) compared with normal (N) tissues in SKCM (melanoma) and BRCA (breast cancer). (C) Expression of SLC3A2 alongside two other tumor associated antigens (EGFR and HER2) in normal breast and skin samples. Mann-Whitney U t-test was performed. ns=non-significant; *p<0.05; **p<0.01; ***p<0.001; ****p<0.0001. See also online supplemental figure S3 and online supplemental materials and methods.

It was previously reported that the murine SF-25 clone demonstrated normal human tissue binding against a subpopulation of cells in the distal tubules of the kidney.^2^ Using RNA-sequencing raw data from The Cancer Genome Atlas (TCGA) and the Genome Tissue Expression (GTex) we examined the expression of SLC3A2 in malignant vs normal tissues across a range of human cancers (figure 3A and online supplemental figure S3). Tumors were selected for analysis when adequate equivalent normal tissue data were available. For the majority of tumors assessed, significantly higher levels of SLC3A2 expression were observed in malignant vs normal tissues, most notably in colorectal, breast, genitourinary cancers and cutaneous melanomas (figure 3A). When considering primary vs metastatic tumors, analysis was hampered by the paucity of metastatic tumors covered by TCGA and GTex. Data were analyzed for melanoma (SKCM) and breast cancer (BRCA) where notably only melanoma had a large resource of metastatic disease available (figure 3B). These data showed that SLC3A2 overexpression was maintained in metastatic deposits of melanomas (figure 3B). True TAAs, limited only to tumor tissue, are very hard to come by in solid tumor oncology. Instead, we develop targeted immune therapy directed against antigens where expression is largely higher in malignant vs normal tissue. To explore the levels of SLC3A2 in relation to established TAAs, we compared SLC3A2 expression levels with those recorded for HER-1 and HER-2 in normal skin and breast tissues. HER-1 and HER-2 were selected as key examples of antigens targeted safely and successfully in the oncology clinic with immune therapy strategies including antibody and cell therapy approaches. SLC3A2 expression, in these normal tissues, is similar or lower than HER-1 and HER-2 expression levels (figure 3C).

**Figure 4:**
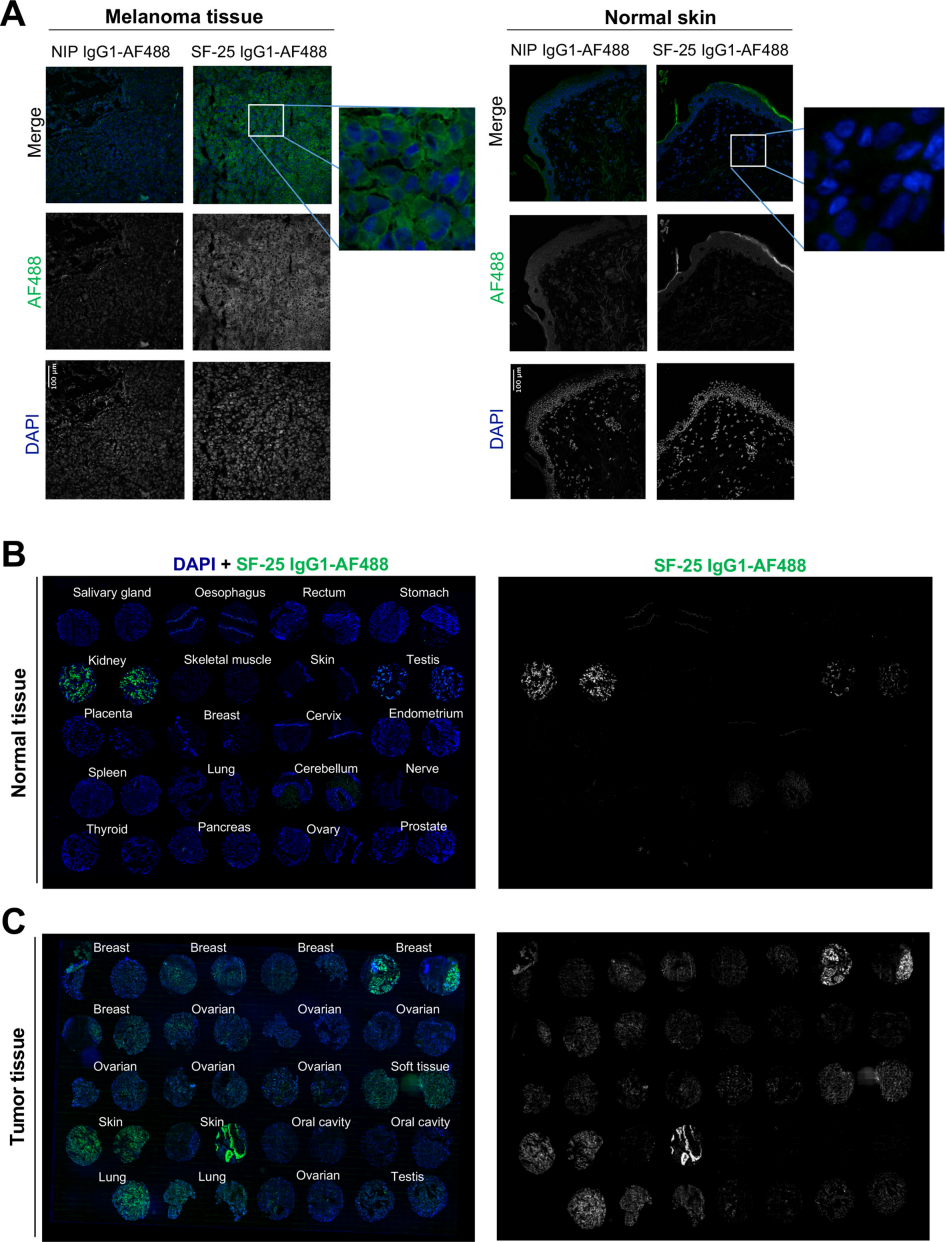
Immunofluorescence evaluations confirm SF-25 antibody reactivity with several tumor tissues versus low/restricted reactivity with normal tissues. (A) melanoma (left) and normal skin (right) frozen tissue sections were stained with SF-25 IgG_1_-AF488 antibody (green) or the negative isotype control NIP IgG_1_-AF488 to detect antigen expression. 4′,6-diamidino-2-phenylindole (DAPI) (blue) was used to reveal the cell nuclei. (B, C) A normal tissue (B) and a cancer specimen tumor microarray (TMA) (C) was stained with SF-25 IgG_1_-AF488 antibody (green) to detect clone reactivity to tumor versus normal tissues. DAPI (blue) was used to reveal the cell nuclei.

We next evaluated the reactivity of the antibody clone against human malignant and non-malignant tissues by immunohistochemistry. Directly labeled SF-25 IgG_1_-AF488 showed reactivity against melanoma but not against normal skin samples (figure 4A). Tissue microarray immunofluorescence analyses showed no/restricted reactivity in most normal tissues. Consistent with previous data, we confirmed kidney binding and revealed lower-level antibody bindings in testis, and at low levels in human cerebellum (figure 4B). In contrast, robust staining was observed in human malignant tissues, including melanoma, breast, ovarian, testis and soft tissue cancers (figure 4C). Our transcriptomic and immunohistochemical analyses (figures 3 and 4) are consistent with previous reports of SF-25 antibody reactivity against several cancer types versus normal tissues.

To investigate reports of SLC3A2 expression in human peripheral blood mononuclear cells (PBMCs) we stained PMBCs with the MEM-108 anti-CD98hc monoclonal antibody, demonstrating binding that was significantly increased by activation of PBMCs with Phytohemagglutinin (online supplemental figure S4).

The protein derivative of SLC3A2 is an established heterodimerization partner for multiple solute carriers. SLC3A2/CD98hc and two L-type amino acid transport binding partners, LAT1 and ascAT1 (derived from SLC7A5 and SLC7A10, respectively), were analyzed independently as prognostic markers in bladder, breast, cervical, lung, renal and head and neck cancers. Survival over 10 years from diagnosis was assessed by the Kaplan-Meier method for high and low expressing tumors (online supplemental figure S5). Diversity in prognostic value of SLC3A2 and two of its many binding partners highlights the breadth of impact potentially associated with targeting this key heterodimerization TAA.

These findings, at the transcriptomic and cell surface proteomic levels, support SLC3A2 as a TAA and confirm reactivity of the human Fc engineered antibody to human tumor tissues of different origins.

### SF-25 IgE activates immune cells through the FcεRI and demonstrates tumor cell cytotoxicity in vitro

Since IgE antibodies may offer an alternative immunotherapy approach for solid tumors, we investigated the antitumor functions of the engineered SF-25 IgE antibody in vitro.

**Figure 5:**
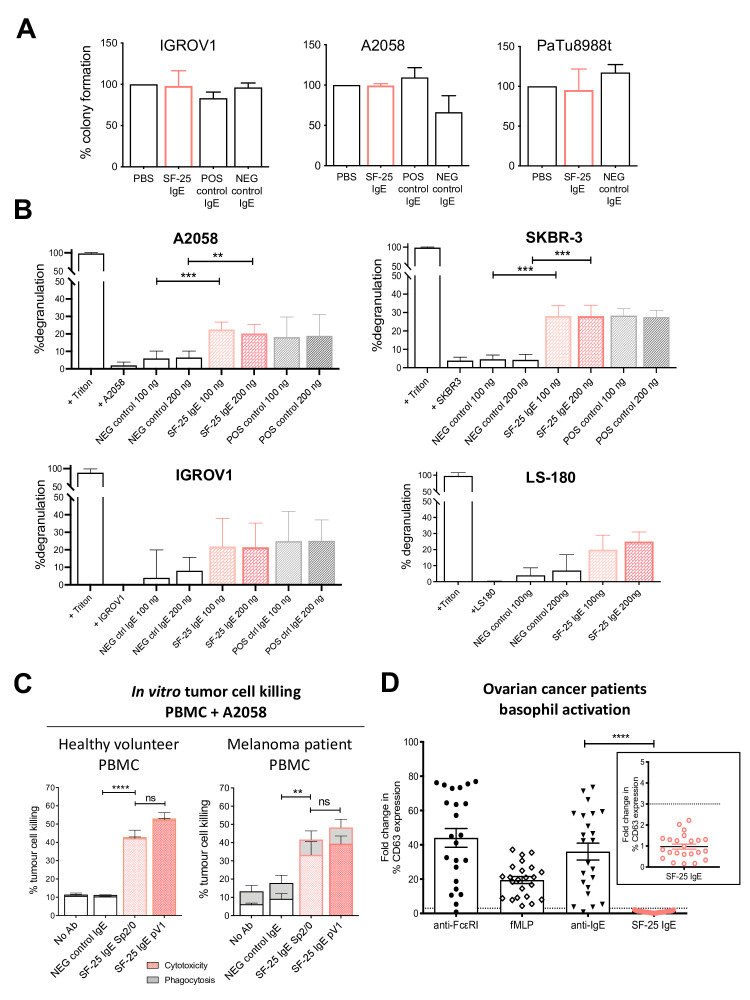
SF-25 IgE can trigger Fc-mediated effector functions. (A) SF-25 IgE did not impair the formation of human ovarian IGROV1, melanoma A2058 or pancreatic PaTu8988t cancer cell colonies. An antimelanoma (CSPG4) IgE was used as negative control with IGROV1 and PaTu8988t cells and as a positive control with A2058 melanoma cells. Antifolate receptor alpha (FRα) antibody MOv18 IgE had no effect on FRα-expressing IGROV1 ovarian cancer or on FRα-negative A2058 melanoma cells. Data represent average ±SD of n=2 independent experiments. (B, C) SF-25 IgE crosslinked on immune effector cells by target antigen-expressing cancer cells triggered effector functions: (B) degranulation of rat basophilic leukemia (RBL-SX38) cells expressing human FcεRI measured by β-hexosaminidase release: SF-25 IgE was cross-linked with SF-25 antigen-expressing melanoma A2058 (n=5), breast cancer SKBR-3 (n=4), ovarian cancer IGROV1 (n=2) or colorectal cancer LS-180 cells (n=2). CSPG4 and MOv18 IgE antibodies were used as controls; Triton treatment represented total β-hexosaminidase release controls (average ±SD of independent experiments). Welch’s test was performed. **p<0.01; ***p<0.001. (C) Healthy volunteer (left) and melanoma patient (right) peripheral blood mononuclear cells (PBMCs) activated by SF-25 IgE to mediate ADCC of A2058 melanoma cells. MOv18 served as negative control (% tumor cell killing ±SEM of n=4 independent experiments). (D) the propensity of SF-25 IgE to mediate activation on human basophils ex vivo was evaluated using the basophil activation test (BAT), in unfractionated peripheral blood of cancer patients, by measuring upregulation of CD63. While IgE:FcεRI- (anti-FcεRI, anti-IgE) and non-IgE (fMLP) mediated basophil activation (fold change in the percentage of CD63-positive cells) in a cohort of n=23 ovarian cancer patients, SF-25 IgE stimulation led to NO basophil activation (NO increase of CD63-positive cells) above background set as threefold change (D, inset). Each value corresponds to a patient sample (n=23 ovarian cancer patients). Mann-Whitney U t-test was performed. ns=non-significant; **p<0.01; ***p<0.001; ****p<0.0001 (see also online supplemental figure S6).

To determine direct effects of SF-25 IgE on target cancer cells, colony formation assays with A2058, IGROV1 and PaTu8988t cells were performed. SF-25 IgE did not impair the ability of cancer cells to form colonies (figure 5A).

We next investigated whether SF-25 IgE could trigger Fc-mediated effector functions through the high affinity Fc receptor FcεRI. Mast cells and basophils are known to express FcεRI and to participate in parasite clearance through IgE.^19^ The rat basophilic leukemia RBL SX-38 in vitro mast cell model expressing human FcεRI, was used to examine the Fc-mediated biological activities of SF-25 IgE.^20^ We investigated the Fc-mediated functions of SF-25 IgE to trigger mast cell degranulation when cross-linked by multiple copies of its target antigen expressed on the surface of cancer cell lines A2058, IGROV1, SKBR-3 and LS-180. Degranulation, measured by β-hexosaminidase release, in the presence of non-specific control IgE was minimal, while SF-25 IgE triggered significant mast cell degranulation in the presence of different target expressing cancer cells (figure 5B). ADCC was measured in coculture experiments using human effector cell and target cell fluorescence reporters (online supplemental figure S6A, B). In the presence of healthy volunteer or melanoma patient PBMCs, SF-25 IgE engendered significant A2058 melanoma cell killing above non-specific isotype IgE or no antibody controls (figure 5C).

In vitro, SF-25 IgE did not affect proliferation or the clonogenic ability of cancer cells. The antibody exerted Fc-mediated effector functions via the high affinity FcεRI receptor. When cross-linked by multiple copies of an antigen expressed on the target cell surface, SF-25 IgE mediated specific mast cell degranulation and triggered cytotoxicity of cancer cells by both healthy volunteer and cancer patient immune cells.

### SF-25 IgE does not mediate basophil degranulation in a whole cancer patient blood assay ex vivo

An IgE antibody introduced in the human circulation could bind to FcεRI-expressing basophils in the blood. If basophil bound IgE is cross-linked by signals such as multivalent soluble circulating antigen or by antibodies recognizing circulating multivalent antigen, this could trigger basophil degranulation, potential hypersensitivity and the onset of systemic anaphylaxis. We therefore evaluated the potential of SF-25 IgE to trigger basophil degranulation in human cancer patient blood using the basophil activation test (BAT). The BAT assay is used to monitor for hypersensitivity to the first-in-class IgE immunotherapeutic as part of its clinical development.^9 15 21^ Whole blood samples from ovarian cancer patients were incubated with either stimulation controls: anti-FcεRI antibody to crosslink the IgE receptor, fMLP (*N*-Formylmethionyl-leucyl-phenylalanine), a polyclonal activator of human basophils, or polyclonal anti-human IgE to cross-link endogenous IgEs already bound to the surface of human blood basophils. Basophil populations were identified by expression of CCR3 (online supplemental figure S5A). Basophil activation was evaluated by detection of CD63 cell surface expression, a marker normally absent in resting basophils (online supplemental figure S6C, D). Marked levels of basophil activation were observed when ovarian cancer whole blood samples were incubated with the three positive stimulation controls. These data confirmed propensity for patient basophil activation by both IgE and non-IgE-mediated mechanisms (figure 5D). In contrast, the incubation of blood with SF-25 IgE did not lead to basophil activation above a threefold threshold (figure 5D inset) in any of the 23 cancer patient samples tested.

These findings suggest that SF-25 IgE could not trigger basophil activation in a functional ex vivo assay, demonstrating no hypersensitivity reaction in cancer patient whole blood. These findings provide early evidence to support safe administration of this antibody to patients.

### A SLC3A2 specific CAR derived from the SF-25 antibody clone demonstrates tumor cell cytotoxicity in vitro

**Figure 6:**
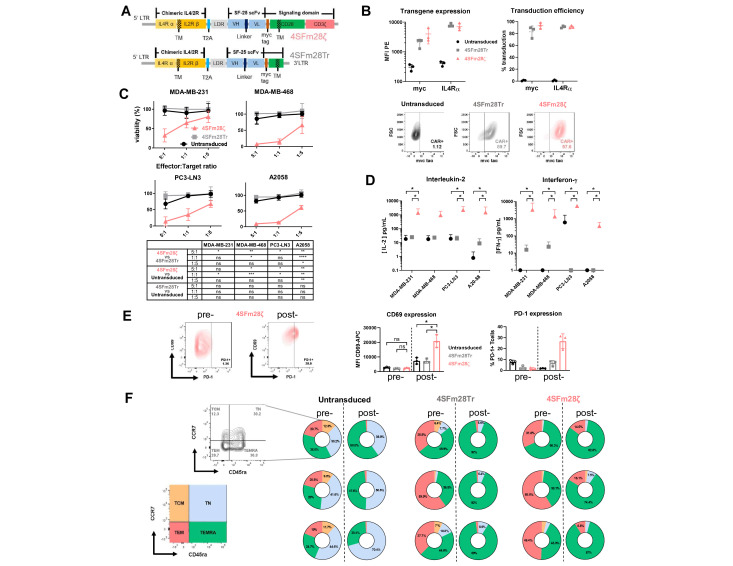
4SFm28ζ CAR T cells effectively target adherent cancer cell lines in vitro. (A) Plasmid maps of the second generation 4SFm28ζ CAR construct (top panel) and 4SFm28Tr control construct (bottom panel). (B) Transgene expression level, expressed as MFI, and transduction efficiency, as percentage transduction, of untransduced (black circles), 4SFm28Tr (gray squares) or 4SFm28ζ (red triangles) CAR constructs. cells were probed after 10 days in culture for the myc-tag, incorporated in the extracellular domain of the constructs, with the anti-MYC tag antibody (9E10). MFI reported as average ±SD of n=3 independent experiments. (C) cytotoxicity of 4SFm28ζ CAR T cells on four different tumor cell line monolayers. Plots represent the percentage of viable tumor cells after a 24-hour coculture with untransduced (black circles), 4SFm28ζ (red triangles) or 4SFm28Tr (gray squares) T cells from three different donors±SD at three different E:T ratios. (D) Interleukin-2 (bottom left panel) and interferon-gamma (bottom right panel) concentrations in coculture supernatants at 24 hours. Supernatants from untransduced (black circles), 4SFm28Tr (gray squares) and 4SFm28ζ (red triangles) T cells from three different donors±SD. (E) Activation markers (CD69 and PD-1) were probed pre (open symbols) and post (closed symbols) 72-hour coculture on PC3-LN3 monolayers at a 1:2 E:T ratio. Representative dot plots are shown. CD69 expression by MFI preantigen and postantigen exposure on untransduced (black circles), 4SFm28Tr (gray squares) and 4SFm28ζ (red triangles) T cells from three different donors±SD. PD-1 percentage expression is also demonstrated. (F) T cell phenotyping pretarget and post-target cells exposure. representative plot (top left) for untransduced T-cells. Color-coded chart (bottom left) of phenotype quadrants. Pie-charts representing clockwise, the central memory (TCM), naïve (TN), terminally differentiated CD45RA+ (TEMRA) and effector memory (TEM) subsets pre- and post- coculture for untransduced (middle left), 4SFm28Tr (middle right) and 4SFm28ζ (right) live T-cells. n=3, each row represents a single donor. CAR, chimeric antigen receptor; E:T, effector to target; MFI, mean fluorescent intensity. Two-way ANOVA multiple comparison statistical analysis was performed in (C). Mann-Whitney U t-test was performed in (D) and (E). ns=non significant; *p<0.05; **p<0.01; ***p<0.001; ****p<0.0001.

To generate an SLC3A2-specific CAR, we first sequenced the variable regions of the heavy and light chain of the SF-25 clone and performed sequence modifications to the framework 1 region of both chains. The resultant single-chain variable fragments (scFv) were fused to a modified CD28 hinge containing a myc-tag, CD28 transmembrane and costimulatory intracellular domains and CD3ζ stimulatory domain (4SFm28ζ).^22^ A control CAR, truncated at the intracellular domain of CD28, was also engineered (4SFm28Tr). Retroviral vector cassettes were generated with the 4αβ chimeric cytokine receptor upstream of the CARs separated by a T2A sequence (figure 6A). The 4αβ (interleukin 4, IL4/2R) enables selective expansion and enrichment of CAR-positive T cells after transduction through delivery of an IL-2 binding signal in response to IL-4.^23^ After 10 days in IL-4 supplemented culture, robust CAR expression was seen (figure 6B).

High (melanoma—A2058), middle (prostate—PC3-LN3, breast—MDA-MB-468) and low (breast—MDA-MB-231) SF-25 expressing cell lines (figure 1C) were used for in vitro cytotoxicity analysis at effector to target (E:T) ratios of 5:1, 1:1 and 1:5. Significant CAR specific cytotoxicity was demonstrated at 24 hours at all coculture densities for the A2058 and MDA-MB-468 cell lines. For PC3-LN3 4SFm28ζ cytotoxicity was significant compared with 4SFm28Tr at the 5:1 E:T ratio and against untransduced T cells at 5:1 and 1:1. For the low density MDA-MB-231 cell line significantly superior 4SFm28ζ cytotoxicity was seen vs 4SFm28Tr at E:T 5:1 and untransduced T cells at E:T 1:1 (figure 6C). Culture supernatants probed for and IL-2 demonstrated donor variability. At an E:T of 1:1 at 24 hours Il-2 levels were significantly higher for 4SFm28ζ compared with both controls against PC3-LN3, MDA-MB-231 and A2058. Interferon gamma was raised for 4SFm28ζ compared with controls in all cocultures (figure 6D). These data demonstrate signaling CAR T restricted antigen specific in vitro efficacy of SF-25 derived CAR T cells.

Using coculture at an E:T ration of 1:2 on the prostate cancer monolayer PC3-LN3 4SFm28ζ, 4SFm28Tr and untransduced T cells were probed for phenotype and markers of activation (CD69 and PD-1) prior to and after exposure to monolayers. MFI of CD69 significantly increased on 4SFm28ζ CAR T cells after exposure to antigen compared with controls. Accordingly, PD-1 percentage increased post antigen exposure for the signaling CAR (figure 6E). On exposure to PC3-LN3, activation marker levels increased for the 4SFm28ζ signaling CAR only, commensurate with antigen specific activation (figure 6E). Viral transduction alone (for 4SFm28ζ and 4SFm28Tr) resulted in loss of naïve populations and expansion of both T effector memory (TEM) and T effector memory-RA (TEMRA) populations compared with the activated but untransduced controls (figure 6F). Allowing for the natural variability in donors, the impact of engagement of antigen by CAR T cells resulted in further enrichment of TEMRA (figure 6E). These data demonstrate robust, antigen specific, activation of 4SFm28ζ CAR T cells.

### SF-25 IgE and derivative CAR immunotherapies demonstrate in vivo activity

**Figure 7:**
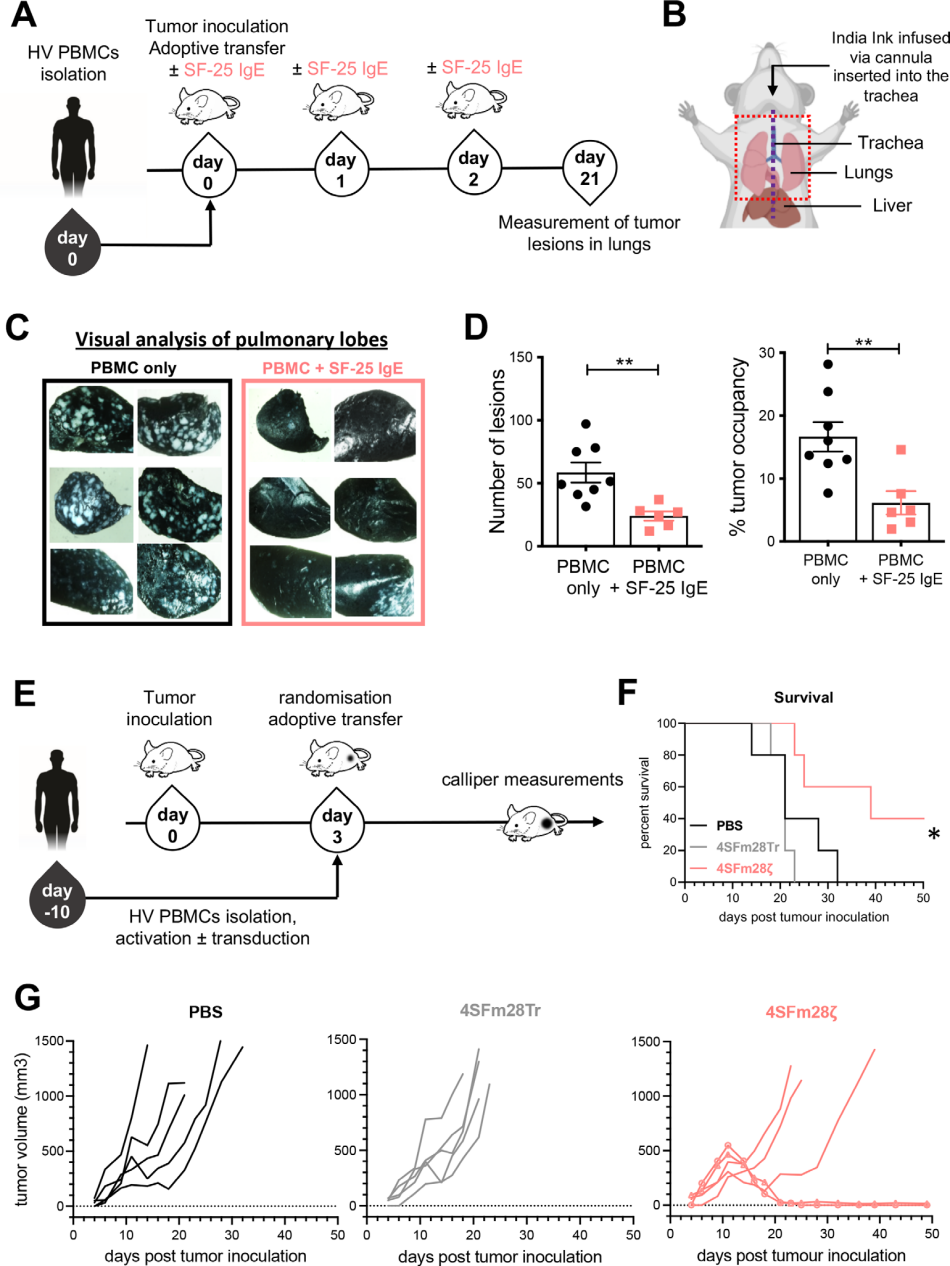
SF-25 IgE and SF-25-based CAR T cells restrict tumor growth in vivo. (A) Schematic representation of the experimental timeline for investigating SF-25 IgE effects on the development of LS-180 cancer lesions in the lungs of NSG mice. (B) Diagram of necropsy procedure for lung removal. The mid-line incision to expose the trachea is depicted with a purple dotted line. The black arrow shows how the Indian ink is infused into the lungs through the tracheal cannula. Lungs were removed en bloc from the thoracic cavity (red dotted line). (C) Representative images of lungs extracted from control-treated and SF-25 IgE-treated animals after staining of tumor-bearing tissues with India ink and subsequent analysis under an inverted light microscope. (D) Scatter plots of the number of tumor lesions (on the right) and of the percentage of tumor occupancy (left) in mouse lungs (each dot represents values from one mouse, average measurement ±SEM of each experimental group (PBMCs: n=8; SF-25 IgE +PBMCs: n=6) and data from two independent experiments with two human PBMC donors; non-parametric t-test; tumor occupancy. **p-value 0.0061, number of metastases **p-value 0.0028. (E) Schematic representation of the experimental timeline employed to investigate SF-25 based CAR effects on tumor growth in a PC3-LN3-ffLucTdTomato subcutaneous model in NSG mice. Total bioluminescence efflux (photons per second) was measured to verify tumor engraftment and randomize the mice at day 3. (F) Survival curves for the three different groups, mice have been culled when tumors were about to reach 1500 mm^3^ or had lost 15% of their weight at day 0 (statistical analysis performed with Mantel-Cox log-rank test; *p value 0.0189). (G) Individual tumor growth curves as measured with calipers in PBS treated (left), truncated CAR T cells (middle) and SF-25 second generation CAR T cells (right panel). The two long-term remission mice are highlighted with round and square symbols on the curve (see also online supplemental figure S7). CAR, chimeric antigen receptor; PBMCs, peripheral blood mononuclear cells. Mouse image used courtesy of MCKIBILLO.

Immunodeficient NSG mice were intravenously injected with human LS-180 colorectal cancer cells which led to the development of cancer lesions in animal lungs. Freshly isolated human PBMCs were adoptively transferred in the presence or absence of SF-25 IgE. Antibody treatment alone was readministered twice more (figure 7A). After 3 weeks, tumor load in the lungs of mice was measured (figure 7B). Analyses demonstrated significantly restricted tumor growth in SF-25 IgE-treated mice, both in relation to the number of metastatic foci (p=0.0028) and with regard to the area of tumor occupancy in animal lungs (p=0.0061) compared with PBMC treated controls (figure 7C, D).

For CAR T in vivo efficacy testing, PC3-LN3-luciferase subcutaneous xenografts were established in NSG mice. Tumor engraftment was confirmed by bioluminescence imaging. Tumor growth was monitored by caliper measurements as central tumor necrosis limits bioluminescence accuracy. Tail vein injections of 4SFm28ζ and 4SFm28Tr CAR T cells were undertaken 3 days after tumor inoculation (figure 7E). A significant increase in overall survival was seen in the 4SFm28ζ group (p=0.0208) (figure 7F). Of five, two treated mice demonstrated tumor eradication after initial growth, with one showing initial response followed by tumor escape (figure 7G). The animals displayed no signs of weight loss or manifest any symptoms of cytokine release syndrome or graft-versus-host disease (online supplemental figure S7).

These findings collectively support the functional capability of novel immunotherapies targeting SLC3A2 to restrict the growth of tumors and improve survival in vivo in the absence of overt toxic effects.

## Discussion

We have identified the solute carrier family member SLC3A2 as the target of the SF-25 antibody clone. The bioinformatics approach described here constitutes a novel approach to identify target proteins for an antibody with unknown antigen specificity. A similar comparison method^24^ has been applied once for identifying an aptamer’s target.^25^ The SLC3A2 protein CD98hc is expressed at high levels on several solid tumors. Normal tissue distribution is restricted to kidney, testis and cerebellum. We have demonstrated the utility of SLC3A2/CD98hc targeting with two highly current immunotherapy approaches. First, human IgE class engineering of the SF-25 mouse clone and second, integration of a derivative scFv into a CAR T cell approach.

The protein product of SLC3A2 (initially known as 4F2 cell-surface heavy chain (4F2hc))^26 27^ was identified as the heavy chain subunit of CD98 complexes (CD98hc).^28^ CD98hc is a component of cell surface heterodimeric complexes, stabilized by disulfide bonds, with several putative light chain subunits,^29 30^ including the L-type amino acid transporters LAT1 (SLC7A5),^31^ LAT2 (SLC7A8),^32^ y+LAT1 (SLC7A7),^33^ y+LAT2 (SLC7A6),^34^ ascAT1 (SLC7A10),^35^ and the cystine/glutamate antiporter xCT (SLC7A11).^36^ Each of these complexes allows specific solutes to cross the plasma membrane, the light subunit conferring the solute specificity to the complex.^29^ In concordance, our bioinformatics analysis showed that SLC3A2 and SLC7A5 expression highly correlated with binding of the SF-25 antibody. This suggests that SF-25 may be specifically, or preferentially, interacting with the CD98hc-LAT1 heterodimer. Additionally, CD98hc acts as a chaperone protein enabling translocation of heterodimerization partners from the endoplasmic reticulum to the cell surface. The resulting amino acid trafficking is central to cell functionality, providing essential amino acids for protein, vitamin and nucleotide synthesis.^28 37 38^

CD98hc has been demonstrated to stabilize the glucose transporter GLUT1,^39^ and to interact with galectin-3, ICAM-1, CD9, and integrins β1a, β3 and β4, suggesting a broader role in tissue architecture.^40–44^ It is directly involved in pathways leading to ER-stress responses,^45^ oxidative stress responses,^46 47^ B and T cell activation,^48 49^ cell fusion,^40^ mechanotransduction,^50^ angiogenesis,^51^ cell survival and migration,^52^ and cell proliferation.^48 53 54^ Highly proliferative tissues, in health and disease, overexpress CD98hc. It has been directly linked to tumorigenesis by mediating β1 integrin signaling^52^ and indirectly by affecting the mTOR activity^54^ through its associated light chain amino acid transporters.^55^

The potential to capitalize on CD98hc expression and functional importance to highly metabolic tumors has been established.^56–69^ A role in resistance to chemotherapy,^70 71^ radiotherapy,^72^ and to T cell-mediated killing through inhibition of ferroptosis,^73^ together highlight potential for integrating targeting with established therapeutic modalities to overcome resistance. Recent immunotherapy and theranostic approaches have been developed.^74–76^

In this study, we report the engineering, production, purification and functional evaluations of two immunotherapy approaches based on SF-25. SF-25 IgE demonstrates specific antitumor activity in vitro and in vivo. Our results are consistent with previous findings with our first in class IgE antibody MOv18, specific for the TAA folate receptor alpha (FRα). MOv18 is in phase 1 clinical testing with early data demonstrating safety and signs of biological activity in patients with ovarian cancer.^8 9 77^ We showed that SF-25 IgE could restrict the growth of tumor lesions in the lung of mice when administered with human immune effector cells. Administration of human immune effector cells was necessary in murine models to study the ability of SF-25 IgE to engender Fc Receptor interactions as the human IgE Fc does not cross-react with mouse Fc receptors. Employing a severely immunocompromised animal model could underestimate the antitumor activity of the IgE tested since the absence of a self-replenishing supply of effector cells, a lack of mature mast cells and a short human PBMC lifespan in immunocompromised mice all limit the time frame for an antibody to exert effector functions in an in vivo rodent model.^78^ Future studies could address this limitation through cytokine or growth factors supplementation to enhance the survival of specific human immune cell populations.^79 80^

By interrogating a basophil activation (BAT) test, we demonstrated that SF-25 IgE is unlikely to elicit a type I hypersensitivity reaction in cancer patient blood. These findings support early preclinical safety evaluations for this antibody. The BAT is employed alongside clinical observations, and biological parameters, to monitor and potentially predict safe administration of IgE immunotherapies.^15^ For SF-25 IgE, further ex vivo BAT assays using different SLC3A2^+^ cancer patient cohorts will confirm lack of basophil activation and provide confidence in future safe administration to humans.

Our second immunotherapy approach, with a 4SFm28ζ CAR T cells, has broad potential for clinical translation. Loss of cytotoxicity below a 1:1 E:T ratio highlights the ‘tunability’ of CAR for this target. Expression of CD98hc on activated T cells has been reported.^81^ This raises the possibility that ‘fratricide’ of CD98hc targeting CAR T cells could occur during manufacturing. Nonetheless, we were able to consistently generate sufficient CAR-positive T cells and there was no suggestion of increased enrichment for the 4SFm28ζ transduced cells, which one would expect if there was significant stimulation through the chimeric receptor during expansion. One Uni-CAR approach targeting CD98hc, based on the monoclonal antibody MEM-108, recently demonstrated no fratricide during coculture with tumor monolayers, despite transient expression on activation. The authors concluded that the antigen density on target cells was far higher than the CAR T cells with the threshold for cytotoxicity higher than the level of T cell expression of CD98hc.^74^ The upregulation of CD98hc in both activated immune cells and tumor cells suggest that these cells compete for the same metabolites as a source of energy. Targeting SLC3A2 on cancer cells could enhance the ability of immune cells to function in the tumor microenvironment.^82^ Loss of target expression is a key mechanism of acquired resistance to CAR T cell therapies in the clinic.^83^ In the case of SLC3A2, we observed that its knock-out in A2058 cancer cells stopped their in vitro proliferation, which is consistent with previously reported data both in vitro and in vivo.^66^ As a consequence, antigen loss is unlikely to be a resistance mechanism to SLC3A2 targeting as it would result in impaired cell proliferation in cancer cells. Future studies combining this CD98hc specific CAR with additional engineering strategies could further enhance the potential of 4SFm28ζ as a translatable cancer therapeutic.

## Conclusion

Immune metabolism is a nascent and fertile area of therapeutic discovery. The preferential transport and utilization of key solutes by malignant cells in a tumor microenvironment is both key to tumor progression and resistance to immune-mediated killing. The role of SLC3A2/CD98hc in transporting and stabilizing multiple solute transporters and cell–cell adhesion molecules makes it a highly attractive candidate for immune therapy development. Here, we have shown its utility for novel antibody class and CAR T cell targeting and provide early evaluations of safety. Our findings form the basis for developing new treatment options for aggressive cancers and contribute to the short but expanding panel of promising target antigens for novel immunotherapies.

## Data Availability

All data relevant to the study are included in the article or uploaded as online supplemental information. Data and material supporting this study are included in the article or uploaded as online supplemental information.
