# Supplementary material for: Immunotherapy using IgE or CAR T cells for cancers expressing the tumor antigen SLC3A2
Source: J Immunother Cancer. 2021 Jun 10;9(6):e002140. doi: 10.1136/jitc-2020-002140 (PMC8194339; doi:10.1136/jitc-2020-002140)
Supplement: Supplementary data [file jitc-2020-002140supp001.pdf]

## SUPPLEMENTAL MATERIALS & METHODS

### Human blood samples

Human samples were collected with informed written consent. Melanoma patient blood samples were collected as part of studies conducted at King's College London, Guy's and St Thomas' NHS Foundation Trust (08/H0804/139 approved by London Bridge NRES committee and 16/LO/0366 approved by London-Central NRES Committee); Ovarian cancer patient blood samples were collected as part of a study conducted at King's College London, Guy's and St Thomas' NHS Foundation Trust (09/H0804/45). Peripheral venous blood was collected in BD Vacutainer™ Hemogard Closure Plastic K2-EDTA Tubes (BD, Wokingham,UK). Peripheral blood was also obtained through the UK National Health System (NHS) Blood and Transplant system from anonymous donor leukocyte cones.

### PBMC isolation from blood samples

Equal volumes of blood and 2% FCS/2 mM EDTA were gently mixed to a final volume of 30 mL and gently pipetted on top of 15 mL of Ficoll-Paque™ PLUS density gradient in a 50 mL conical tube. The tube was then centrifuged at 1200 x g with slow acceleration and no brake at room temperature (RT) for 20 minutes. The plasma interface was collected and washed with PBS at 600 x g at 4°C for 10 minutes. The erythrocytes present in the sample were lysed with RBC lysis buffer for 5 minutes at RT, followed by washing with PBS + 2% FCS/2 mM EDTA.

### Cell lines

IGROV1 ovarian cancer cells were a gift from Prof. Silvana Canevari (Istituto Nazionale dei Tumori, Milano, Italy). PaTu-8988-T and PANC-1 human pancreatic cancer cell line were kindly provided by Dr Debashis Sarker at the department of Research Oncology, School of Cancer and Pharmaceutical



Pellizzari G, et al. J Immunother Cancer 2021; 9:e002140. doi: 10.1136/jitc-2020-002140

Sciences, King's College London. The rat basophilic leukemia RBL SX-38 cells transfected to stably express the human FcεRI $\alpha\beta\gamma_2$ was kindly provided by Professor J.P. Kinet (Harvard University, Boston, USA). The PC3-LN3 (PL) cell line was kindly provided by Professor Sue Eccles (Institute of Cancer Research, Sutton, UK). All other cancer cell lines were sourced from ATCC. Expi 293-F human embryonic kidney cells were from ThermoFisher. Cell culture media for cell lines were supplemented with 10% fetal calf serum (FCS) (v/v), 100 U/mL penicillin and 100 U/mL streptomycin. PBMCs and T cells were cultured in RPMI1640 plus 5% human serum. Adherent cell lines were passaged once they reached 80-90% confluence by detachment in 0.5% Trypsin/0.53 M EDTA at 37°C for 5 minutes, washed and plated in fresh media.

**SF-25 antigen immunoprecipitation**

Pellets containing 20 to $50x10^6$ target expressing MDA-MB-231, A2058 or MDA-MB-468 cells were resuspended in 1.75mL lysis buffer (PBS, 0.1% Tween20, 1X Halt™ protease inhibitors cocktail – Thermo Scientific) in a 15mL tube, incubated at 4ºC on a roller and vortexed for 20 minutes. 300µL ProteinA Dynabeads® (Invitrogen) were prepared with 100µg SF-25 humanized IgG1 in 800µL Binding Buffer as per manufacturer's protocol. Cell lysates were centrifuged for 10 minutes at 4500rcf at 4ºC and supernatants were transferred to the washed SF-25 Dynabeads®. Beads with immunoprecipitated fraction were placed in 30µL of Elution Buffer and 10µL of 4X LDS sample buffer (Invitrogen) and stored at -20ºC. Samples were thawed, beads were concentrated on a magnet, supernatant were transferred to a 1.5mL microcentrifuge tube and β-mercapto-Ethanol was added to a final concentration of 5% (v/v). Samples were incubated for 10 minutes at 95ºC before being resolved on a 4-12% gradient NuPAGE™ gel (Invitrogen) at 200 Volts in MOPS buffer. Migration was stopped when Coomassie G250 reached the bottom of the gel. The gel was fixed in 7% Acetic Acid/40% methanol (v/v) for 30 minutes at room temperature and stained with a 1X solution of colloidal Brilliant Blue G with 20% methanol for 1 hour at room temperature. The gel was first destained for 5 minutes with 7% Acetic Acid and

25% methanol and then overnight with 2% acetic Acid and 25% methanol and stored in distilled water. Bands of interest were cut out and sent to Aulesa Biosciences for mass spectrometry analysis.

**SF-25 antigen: Mass spectrometry analysis**

Proteins in gel slices were reduced (DTT), alkylated (iodoacetamide) and digested overnight with trypsin. Peptides within the tryptic digests were fractionated using an Ultimate 3000 nano-LC system in line with an Orbitrap Fusion Tribrid mass spectrometer (Thermo Scientific). In brief, peptides in 1% (v/v) formic acid were injected onto an Acclaim PepMap C18 nano-trap column (Thermo Scientific). After washing with 0.5% (v/v) acetonitrile 0.1% (v/v) formic acid peptides were resolved on a 250 mm x 75 µm Acclaim PepMap C18 reverse phase analytical column (Thermo Scientific) over a 150 min using 7 gradient segments (1-6% solvent B over 1min, 6-15% B over 58min, 15-32%B over 58min, 32-40%B over 5min, 40-90%B over 1min, held at 90%B for 6min and then reduced to 1%B over 1min) with a flow rate of 300 nL.min$^{-1}$. Solvent A was 0.1% formic acid and Solvent B was aqueous 80% acetonitrile in 0.1% formic acid. Peptides were ionized by nano-electrospray ionization at 2.2 kV using a stainless-steel emitter with an internal diameter of 30 µm (Thermo Scientific) and a capillary temperature of 250°C. All spectra were acquired using an Orbitrap Fusion Tribrid mass spectrometer controlled by Xcalibur 2.0 software (Thermo Scientific) and operated in data-dependent acquisition mode. FTMS1 spectra were collected at a resolution of 120 000 over a scan range (m/z) of 350-1550, with an automatic gain control (AGC) target of 400 000 and a max injection time of 100ms. The Data Dependent mode was set to Cycle Time with 3s between master scans. Precursors were filtered according to charge state (to include charge states 2-7), with monoisotopic precursor selection and using an intensity range of 5E3 to 1E20. Previously interrogated precursors were excluded using a dynamic window (40s +/-10ppm). The MS2 precursors were isolated with a quadrupole mass filter set to a width of 1.6m/z. ITMS2 spectra were collected with an AGC target of 5000, max injection time of 50ms and HCD collision energy of 35%. LC-MS/MS data was processed using Proteome Discoverer

(ThermoFisher Scientific) with database searching against a downloaded FASTA file originating from Uniprot_SwissProt_2019_02. Results were initially visualized within the software and then exported to Excel for further review.

**SLC3A2 differential expression study**

Data for normal and associated tumor tissues were retrieve from the UCSC Toil RNA-seq Recompute dataset.[1] TPM values data from TARGET were filtered out and statistical analysis of SLC3A2, HER1 and HER2 differential expression was assessed by Mann-Whitney test in Graphpad Prism. All tumor samples were paired with the associated normal tissue samples from TCGA and the GTex normal tissues specified below. READ: Rectum Adenocarcinoma (GTex Colon); COAD: Colon Adenocarcinoma (GTex Colon); LIHC: Liver Hepatocellular Carcinoma (GTex Liver); CHOL: Cholangiocarcinoma; ESCA: Esophageal Carcinoma (GTex Esophagus); BLCA: Bladder Urothelial Carcinoma (GTex Bladder); STAD: Stomach Adenocarcinoma (GTex Stomach); OV: Ovarian Serous Cystadenocarcinoma (GTex Ovary); LGG: Brain Low Grade Glioma (GTex Brain); GBM: Glioblastoma Multiforme (GTex Brain); PAAD: Pancreatic Adenocarcinoma (GTex Pancreas); PRAD: Prostate Adenocarcinoma (GTex Prostate); ACC: Adrenocortical Cancer (GTex Adrenal Gland); BRCA: Breast Invasive Carcinoma (Gtex Breast); TGCT: Testicular Germ Cell Tumor (GTex Testis); UCEC: Uterine Corpus Endometrioid Carcinoma (GTex Uterus); UCS: Uterine Carcinosarcoma (GTex Uterus); CESC: Cervical & Endocervical Cancer (GTex Cervix Uteri); THCA: Thyroid Carcinoma (GTex Thyroid); LUAD: Lung Adenocarcinoma (GTex Lung); LUSC: Lung Squamous Cell Carcinoma (GTex Lung); SKCM: Skin Cutaneous Melanoma (GTex Skin); LAML: Liquid Acute Myeloid Leukemia (GTex Blood); DLBC: Diffuse Large B-cell Lymphoma (GTex Blood); HNSC: Head & Neck Squamous Cell Carcinoma; KICH: Kidney Chromophobe (GTex Kidney); KIRP: Kidney Papillary Cell Carcinoma (GTex Kidney); KIRC: Kidney Clear Cell Carcinoma (GTex Kidney).

**Retroviral constructs for CAR expression in PBMCs**

The SF-25 scFv was subcloned into a myc-tag containing 28ζ construct downstream of the 4αβ chimeric cytokine receptor (IL4/2R).[2] The truncated CAR version 4SFm28Tr was generated by PCR by introducing a stop codon and a cloning site after the Lysine in position 3 in the cytoplasmic CD28 fragment. RD114 viral particles were produced by transiently transfecting HEK-293T cells. 1.5x10$^{6}$ cells were plated in a 10cm diameter culture dish in IMDM medium without antibiotics and allowed to grow overnight. Transfection reagents were prepared by gently pipetting 30µL GeneJuice® (Novagen) mammalian cell transfection reagent into 470µL plain IMDM medium (no serum) and incubated for 5 minutes at room temperature. Plasmids were gently added and incubated for 15 minutes before evenly dispensing the transfection reagents dropwise over the 10cm dish. Triple transfections were performed with 3µg RD114, 4.5µg pEQ-Pam3 and 4.5µg of the CAR containing plasmid. Supernatants containing the RD114 viral particles were harvested at 48 and 72 hours post transfection, pooled aliquoted, snap frozen in dry-ice cold ethanol bath and stored at -80$^{o}$C. Stable packaging cell lines were established in HEK293 VECS GalV cell by transducing 1x10$^{5}$ overnight plated cells with 2mL RD114 viral supernatant in 6 well plate. Transduction efficiency in HEK293 VECS GalV cells were assessed by flow cytometry using the 9E10 anti-myc antibody and a polyclonal Goat anti-Mouse IgG PE secondary antibody (Agilent). Cells were transduced with RD114 virus until achieving >95% transduction. For GalV viral production, HEK293 VECS GalV transduced cells were grown in complete DMEM medium without antibiotics in 175cm$^{2}$ culture flask and supernatant was harvested when reaching 90% confluency. GalV virus containing supernatants were aliquoted, snap frozen and stored at -80$^{o}$C.

**Human PBMCs transduction and CAR T cell expansion**

Isolated PBMCs were counted and placed in 6 well non-tissue culture plates at a concentration of 3x10$^{6}$ cells per mL at a maximum of 3mL per well. Polyhydroxyalcanoate (PHA) was used to activate the PBMCs at a final concentration of 5µg/mL. Plates were incubated for 24 hours at 37$^{o}$C and 5% $CO_2$. Interleukin-2 is added at 24 hours to a final concentration of 100U/mL. RetroNectin® (Takara) coating

solution was prepared using polypropylene pipettes by mixing 200µg RetroNectin® with 12mL cold PBS. Non-tissue culture treated 6-well plates were coated by overnight incubation at 4°C. Coating solution was replaced with 2mL GalV virus containing supernatant and incubated at 4°C for 4 hours before being replaced by another 2mL of pre-chilled viral supernatant. At 48 hours post-isolation and 24 hours after Interleukin-2 treatment, $1x10^6$ activated PBMCs were added to each well in 500µL RPMI 5% human serum. Human interleukin-2 and interleukin-4 (R&D systems) were added at a final concentration of 100U/mL and 30ng/mL respectively. Fresh RPMI with 5% human serum containing interleukin-2 (untransduced cells) or interleukin-4 (4αβ transduced cells) was added every 2 days during the expansion phase. Enrichment of CAR positive T cells was assessed by Flow Cytometry on a Fortessa cytometer at days 10 using the 9E10 anti-myc antibody. Analysis of flow cytometry data was performed by FlowJo (TreeStar Inc) software.

**Tumor cell and CAR T cell coculture assays**

Tumor cells were seeded at $5.10^4$ tumor cells per well in 48-well plate in 200µL culture medium without antibiotics and allowed to form monolayers over 24 hours at 37°C in 5% $CO_2$. CAR T cells were washed in PBS and $25.10^4$, $5.10^4$ or $1.10^4$ T cells were added in 100µL culture medium to the monolayers respectively resulting in a 5:1, 1:1 and 1:5 Effector T cell:Target cell ratio. After a 24-hour coculture, 200µL of supernatant was harvested and stored at -20°C for further cytokine measurement. Monolayers viability was assessed immediately as detailed in the main text Methods.

**Cytokine detection after coculture**

Supernatants from cocultures were thawed and analyzed using human Interferon-γ (R&D systems) and human Interleukin-2 (Invitrogen) ELISA kits as described by manufacturer. Supernatants were diluted from 15 to 45-fold to fit within the standard curves. Cytokine levels were plotted using Graphpad Prism.

**Phenotype and activation analysis**

At day 14-post viral transduction, T cells pre-coculture were counted, washed in 2mL FACS buffer and 25.10$^4$ cells were stained either with a cocktail of anti-human CD69-APC (FN50 Biolegend) / anti-human PD-1-PE (EH12.2H7 Biolegend) / antihuman CD3-FITC ; anti-human CD45ra-APC (JS-83 Invitrogen) / anti-human CD197(CCR7)-PE (3D12 Invitrogen) / anti-human CD3-FITC (UCHT1 Biolegend) or corresponding isotypes (MOPC-21 -FITC -APC Biolegend / P3.6.2.8.1-PE Invitrogen) for 20minutes at 4$^o$C. Cells were then washed with 2mL FACS buffer and a Live/Dead staining was performed with 7-AAD reagent as per manufacturer instructions (Cambridge Bioscience). Samples were run on a BD LSRFortessa flow cytometer and live CD3+ cells phenotype and activation pattern were analyzed using FlowJo (TreeStar Inc) Software. Anti-Mouse Ig,κ compensation beads (BD$^{TM}$) were also used. 72 hours cocultures were set the same day in 24-well plates with 1.8x10$^5$ PC3-LN3 overnight-grown monolayers at a 1:2 Effector:Target ratio. After coculture, cells were flushed from 4 wells/construct, pooled and distributed into 3 FACS tubes (2/5$^{th}$ for phenotype labelling, 2/5$^{th}$ for activation labelling and 1/5$^{th}$ for isotype labelling). Same staining, gating strategy and analysis were used in post-coculture conditions as per pre-coculture staining. Analysis of flow cytometry data was performed by FlowJo (TreeStar Inc) software and data were plotted using Graphpad Prism.

**CD98hc Expression on human PBMCs**

Human PBMCs were isolated by density centrifugation as describe in the Methods. Basal expression level was assessed on 1.10$^5$ cells stained with the 1µg anti-CD98hc antibody MEM-108 (Biolegend) for 20 minutes at 4$^o$C, washed with 2mL cold FACS buffer, stained again with goat anti-mouse IgG AlexaFluor647 (Jackson Labs), washed again prior to analysis. Isotype control staining was utilized. Cell surface staining was assessed by Flow Cytometry on a BD LSRFortessa cytometer. PBMCs were then activated or not with PHA as described above. Over 15 days, 1x10$^5$ PBMCs were retrieved from the cell

culture and stained as described above. Analysis of flow cytometry data was performed by FlowJo (TreeStar Inc) software and data were plotted using Graphpad Prism.

**Immunofluorescence study of antigen expression**

SF-25 IgG1 and NIP-IgG1 were directly labelled with Alexa Fluor 488 fluorophore using the Alexa Fluor™ 488 Antibody Labeling Kit (ThermoFisher). Frozen tissues were thawed at room temperature for 10 minutes, allowed to dry and fixed with 4% paraformaldehyde (PFA) for 10 minutes. Once PFA was removed, tissues were washed 3 times with PBS. Sections were covered with Human AB serum for 1 hour at room temperature. Tissues were incubated overnight with SF-25 IgG1-AF488 or NIP-IgG1-AF488 and kept at 4°C in the dark. Sections were washed 3 times in PBS and allowed to dry at RT. ProLong™ Gold Antifade Mountant with DAPI (ThermoFisher) was added, coverslip were applied and slides solidified overnight at room temperature in the dark.

***In vivo* human cancer xenograft model to study SF-25 IgE**

On day 0, NSG mice were injected simultaneously with $5x10^5$ LS-180 colorectal cancer cells, $5x10^6$ PBMCs from a healthy human volunteer and 10mg/kg of SF-25 IgE or 50µl PBS, to a final volume of 200µl. Subsequent injections of antibody/PBS were performed on days 2 and 3. Mice were sacrificed on day 21 by $CO_2$ asphyxiation. Lungs were analyzed for tumor growth in the lungs using the following protocol: the trachea was exposed by performing a mid-line incision from below the diaphragm to the throat and the chest activity opened. An intravenous cannula was inserted into the trachea, and a 10 mL syringe was attached to it. Approximately 3 mL of a solution of 15% (v/v) Indian Ink was then injected into the lungs. The ink-stained lungs were then removed from the thoracic cage, placed in MilliQ water to remove the excess ink and then transferred to Fekete solution for 48 hours. The number of metastatic nodules/lung lobe and the proportional surface occupancy of tumor nodules were calculated. Images were acquired using a Nikon SMZI500 Stereo Microscope (Nikon UK Ltd) with 0.75x

and 1.0x objective. The NIS Elements Basic Research software (Nikon UK Ltd) was used to determine the total surface area of the lung section and to distinguish the white lung metastasis nodules. Data were acquired from two independent experiments using PBMCs from two human donors.

***In vivo* SF-25 CAR T cell in prostate tumor model**

6 to 12 weeks old NSG$^{TM}$ mice have been inoculated with 2.5x10$^5$ PC3-LN3 cells engineered to express ffLuciferase and TdTomato (PL-LT) by subcutaneous (s.c.) injection on the right flank. At Day 3, Bioluminescent imaging (BLI) was performed using Xenogen IVIS imaging system with Living Image software (Xenogen). Mice were injected intra-peritoneally (i.p.) with 200µL of imaging solution (15µg/mL D-Luciferin in PBS), anaesthetized under 2% Isofluorane and imaged after 20 minutes. Mice were distributed in the different groups to ensure minimal variation in mean BLI signal and standard deviation between groups. The same day, 1x10$^7$ CAR T cells were adoptively transferred to mice intra-venously (i.v.) in 200µL PBS. Mice were weighed and their tumors measured by calipers 2 to 3 times a week for the rest of the experiment.

**Lectin blots**

Purified IgE samples (150ng) were reduced with 50mM dithiothreitol and boiled at 95°C for 5 minutes. Samples were run at 150 V on Mini-PROTEAN TGX Gels 4-15% (Bio-Rad Laboratories, Hercules, Calif) and blotted with Trans-Blot Turbo Transfer Pack PVDF (Bio-Rad Laboratories) by using the Trans-Blot Turbo Blotting System (Bio-Rad Laboratories) according to the manufacturer's instructions. The blotted membrane was then cut just above 35kDa to have heavy (50kDa) and light (25kDa) chains in different membranes. The heavy chain membrane was blocked with Carbo-Free Blocking Solution (Vector Laboratories) for 1 hour and then probed with Ricinus communis agglutinin I lectin (RCAI-biotin) [Vector Laboratories] specific for galactose, Aleuria aurantia lectin (AAL-biotin) [Vector Laboratories] specific for fucose, concanavalin A lectin Con-A–biotin) [Vector Laboratories] specific for

mannose, or EBL (Sambucus nigra lectin) [Vector Laboratories] specific for sialic acid at 0.2 µg/mL in Carbo-Free Blocking Solution for 30 minutes. The membranes were then washed 3 times in PBS–Tween 0.05% (T-PBS) and incubated with High Sensitivity Streptavidin-HRP (1:30000; Pierce) for 30 minutes, washed as above, and developed with ECL (Amersham, GE Healthcare). The light chain membrane was blocked with T-PBS and 5% BSA for 1 hour at room temperature and then incubated overnight at 4°C with rabbit anti-human kappa light chain antibody (1:1000 in T-PBS 5% BSA; Abcam, Cambridge, United Kingdom), followed by 3 washes in T- PBS. The membrane was incubated with anti-rabbit IgG horseradish peroxidase antibody (1:2000 in T- PBS and 5% BSA; Cell Signaling Technology, Danvers, Mass) for 1 hour at room temperature, washed as above, and developed with ECL. Densitometric quantification was performed with ImageJ software (National Institutes of Health, Bethesda, Md), and values were normalized by the loading control (kappa light chain).

**Transcript- to binding-level correlation analysis**

This section is divided into three steps, an example of the code used is located at:
https://github.com/ramipod/Antigen-Identification-by-Binding-and-Transcriptomic-Comparison

- TPM dataset generation (TPM averaging)
- Spearman score calculation for a specific binding dataset and the TPM counts (spearman AVG1)
- Spearman scores compilation (Spearman-AVGs)

**Statistical methods**

All statistical analyses were performed using GraphPad™ Prism Software (version 6.0). Error bars represent SD and SEM in *in vitro* and in *ex vivo* evaluations.



Pellizzari G, *et al. J Immunother Cancer* 2021; 9:e002140. doi: 10.1136/jitc-2020-002140

**REFERENCES**

1 Vivian J, Rao AA, Nothaft FA, *et al.* Toil enables reproducible, open source, big biomedical data analyses. Nat. Biotechnol. 2017;**35**:314–6. doi:10.1038/nbt.3772

2 Wilkie S, Burbridge SE, Chiapero-Stanke L, *et al.* Selective expansion of chimeric antigen receptor-targeted T-cells with potent effector function using interleukin-4. *J Biol Chem* 2010;**285**:25538–44. doi:10.1074/jbc.M110.127951

BMJ Publishing Group Limited (BMJ) disclaims all liability and responsibility arising from any reliance placed on this supplemental material which has been supplied by the author(s)


# TableS1

**SF-25 IgG1 immunoprecipitated proteins**

| 42kDa | | | | | 27kDa | | | | |
|---|---|---|---|---|---|---|---|---|---|
| **geneID** | **Score** | **Coverage** | **# Unique Peptides** | **# Peptides** | **geneID** | **Score** | **Coverage** | **# Unique Peptides** | **# Peptides** |
| TPM2 | 595.10 | 42.25 | 5 | 17 | RAB5C | 281.86 | 54.17 | 6 | 9 |
| TPM1 | 405.75 | 21.83 | 1 | 9 | RAB5A | 254.43 | 47.91 | 5 | 8 |
| HLA-A | 381.19 | 38.36 | 2 | 10 | RAB5B | 218.29 | 47.91 | 5 | 8 |
| CALD1 | 355.15 | 14.00 | 14 | 14 | HSPB1 | 159.56 | 50.73 | 7 | 7 |
| TMOD3 | 287.18 | 48.86 | 17 | 17 | TPI1 | 138.62 | 40.16 | 7 | 8 |
| HLA-C | 230.67 | 34.70 | 0 | 9 | PCMT1 | 125.54 | 39.21 | 6 | 6 |
| HLA-C | 212.43 | 22.58 | 0 | 6 | RAB32 | 123.00 | 27.56 | 6 | 6 |
| HLA-C | 191.83 | 27.32 | 0 | 7 | RALB | 118.44 | 27.18 | 4 | 5 |
| ERLIN2 | 183.87 | 30.97 | 9 | 9 | HSD17B10 | 118.12 | 29.12 | 4 | 4 |
| LIMA1 | 182.62 | 16.73 | 12 | 12 | SRPRB | 115.37 | 38.75 | 7 | 7 |
| HLA-A | 174.65 | 21.10 | 0 | 5 | CHCHD3 | 113.81 | 21.59 | 5 | 5 |
| HLA-B | 161.47 | 21.55 | 1 | 6 | RALA | 106.32 | 16.99 | 2 | 3 |
| DBN1 | 151.55 | 14.18 | 9 | 9 | RPL13A | 102.05 | 23.65 | 5 | 5 |
| PDLIM2 | 146.33 | 26.70 | 6 | 6 | ***SLC7A5*** | 96.57 | 8.68 | 3 | 3 |
| HM13 | 140.35 | 23.08 | 8 | 8 | SCO2 | 90.93 | 22.93 | 5 | 5 |
| NSDHL | 135.94 | 31.10 | 9 | 9 | SDHB | 85.91 | 16.79 | 4 | 4 |
| EPCAM | 134.56 | 15.92 | 5 | 5 | PGRMC2 | 84.44 | 8.97 | 2 | 2 |
| GOT2 | 127.12 | 12.33 | 5 | 5 | MRLP9 | 77.25 | 8.24 | 2 | 2 |
| DDRGK1 | 125.83 | 14.01 | 3 | 3 | RAB27A | 72.25 | 15.84 | 4 | 4 |
| STOML2 | 122.72 | 20.79 | 7 | 7 | SSSCA1 | 71.14 | 16.08 | 3 | 3 |
| DHCR7 | 120.25 | 13.26 | 5 | 5 | PTTG1IP | 64.94 | 10 | 1 | 1 |
| GNAI3 | 106.46 | 16.67 | 4 | 5 | TMED7 | 61.82 | 15.63 | 4 | 4 |
| EIF3H | 99.54 | 5.40 | 1 | 1 | GAPDH | 59.89 | 12.84 | 4 | 4 |
| CDS2 | 99.30 | 6.97 | 2 | 2 | PRDX6 | 57.08 | 11.61 | 4 | 4 |
| EGFR | 94.30 | 3.22 | 3 | 3 | ***SLC3A2*** | ***55.80*** | ***3.33*** | ***2*** | ***2*** |
| CAPZA1 | 90.00 | 19.58 | 4 | 4 | SNAP23 | 55.75 | 29.86 | 5 | 5 |
| RCN1 | 82.45 | 8.16 | 3 | 3 | PRDX4 | 55.65 | 16.61 | 4 | 4 |
| ALDOC | 80.02 | 8.24 | 1 | 2 | MRPL16 | 55.55 | 19.92 | 5 | 5 |
| GNAI2 | 75.71 | 10.70 | 2 | 3 | MYADM | 55.48 | 7.14 | 1 | 1 |
| MPZL1 | 67.04 | 12.64 | 3 | 3 | COMT | 53.26 | 23.25 | 4 | 4 |
| HNRNPA3 | 66.70 | 12.70 | 3 | 3 | DSG1 | 52.68 | 2.48 | 2 | 2 |
| DHRS13 | 65.97 | 2.12 | 1 | 1 | RAB12 | 52.01 | 23.77 | 5 | 5 |
| ZC3HAV1 | 65.36 | 1.66 | 1 | 1 | BCAP31 | 51.52 | 11.38 | 3 | 3 |
| SCAMP3 | 64.17 | 8.36 | 2 | 2 | CRIP2 | 50.76 | 23.08 | 2 | 2 |
| ATP1B3 | 63.15 | 8.24 | 2 | 2 | CD97 | 49.95 | 3.95 | 3 | 3 |
| MBOAT7 | 62.63 | 6.14 | 2 | 2 | FLG2 | 48.95 | 0.5 | 1 | 1 |
| TOMM40 | 59.08 | 3.88 | 1 | 1 | CYB561 | 47.80 | 3.98 | 1 | 1 |
| PLEC1 | 58.88 | 0.60 | 2 | 2 | LYZ | 47.18 | 12.84 | 2 | 2 |
| CAPG | 57.18 | 7.18 | 2 | 2 | PEX11B | 47.03 | 13.13 | 3 | 3 |
| GIPC1 | 54.93 | 4.20 | 1 | 1 | AGPAT2 | 46.25 | 12.23 | 3 | 3 |
| HSPB1 | 54.05 | 9.76 | 2 | 2 | SPC25 | 44.74 | 5.36 | 1 | 1 |
| ZMPSTE24 | 53.85 | 10.53 | 5 | 5 | PLSCR1 | 44.57 | 4.09 | 1 | 1 |
| GADPH | 52.57 | 17.61 | 4 | 4 | HAUS2 | 43.54 | 10.21 | 2 | 2 |
| MAGEA4 | 43.66 | 2.21 | 1 | 1 | ETHE1 | 42.97 | 9.06 | 2 | 2 |
| AHSA1 | 42.96 | 2.96 | 1 | 1 | TMED4 | 42.58 | 21.59 | 3 | 4 |
| DAP3 | 39.04 | 13.57 | 4 | 4 | CD151 | 41.94 | 9.88 | 3 | 3 |
| CLEC14A | 38.53 | 1.43 | 1 | 1 | YIPF4 | 41.35 | 3.69 | 1 | 1 |
| RNH1 | 37.94 | 2.60 | 1 | 1 | ITGA6 | 41.27 | 1.95 | 2 | 2 |
| SYPL1 | 37.60 | 4.25 | 1 | 1 | EIF2B2 | 40.25 | 4.59 | 2 | 2 |
| SERPINB1 | 37.05 | 2.90 | 1 | 1 | TMEM41B | 39.33 | 12.37 | 3 | 3 |
| ***SLC7A5*** | 36.39 | 2.76 | 1 | 1 | RRAS2 | 39.21 | 15.69 | 3 | 3 |
| METT5D1 | 35.80 | 3.44 | 1 | 1 | HEXB | 38.47 | 1.8 | 1 | 1 |
| VPS26A | 34.24 | 5.50 | 1 | 1 | BAG2 | 37.27 | 13.74 | 3 | 3 |
| LANCL2 | 33.55 | 6.00 | 2 | 2 | HRNR | 36.68 | 3.47 | 2 | 2 |
| ALG2 | 30.90 | 3.61 | 1 | 1 | BNIP1 | 36.43 | 32.89 | 6 | 6 |
| SGPP1 | 28.93 | 2.95 | 1 | 1 | PYCRL | 35.97 | 21.53 | 4 | 4 |
| OXA1L | 28.41 | 1.84 | 1 | 1 | MTX3 | 33.54 | 7.37 | 2 | 2 |
| MRI1 | 27.83 | 3.52 | 1 | 1 | C7orf50 | 33.43 | 23.71 | 3 | 3 |
| AMY1A | 27.72 | 3.13 | 1 | 1 | CLIC1 | 33.37 | 12.45 | 2 | 2 |
| ACTN1 | 25.85 | 1.35 | 1 | 1 | GAR1 | 31.03 | 4.15 | 1 | 1 |
| MT-ND5 | 24.55 | 1.82 | 1 | 1 | MRPS7 | 30.97 | 10.33 | 2 | 2 |
| CD14 | 23.45 | 5.07 | 1 | 1 | MTX2 | 30.68 | 4.18 | 1 | 1 |
| TMEM43 | 23.26 | 8.25 | 4 | 4 | TSPAN14 | 29.05 | 9.26 | 2 | 2 |
| TMEN175 | 0.00 | 3.17 | 1 | 1 | GOSR2 | 28.69 | 20.75 | 3 | 3 |
| | | | | | DCD | 28.32 | 10 | 1 | 1 |
| | | | | | CASP14 | 28.24 | 3.31 | 1 | 1 |
| | | | | | SYPL1 | 28.24 | 4.25 | 1 | 1 |
| | | | | | TMEM33 | 27.60 | 8.91 | 2 | 2 |
| | | | | | TMEM189 | 27.29 | 5.56 | 1 | 1 |
| | | | | | MRPL24 | 26.09 | 6.02 | 1 | 1 |
| | | | | | DCAF16 | 24.41 | 5.56 | 1 | 1 |
| | | | | | TSPAN6 | 23.59 | 5.31 | 1 | 1 |
| | | | | | S100A8 | 21.29 | 11.83 | 1 | 1 |
| | | | | | GSTO1 | 20.76 | 7.05 | 2 | 2 |
| | | | | | TMEM56 | 20.75 | 4.18 | 1 | 1 |
| | | | | | FHL2 | 20.58 | 10.04 | 2 | 2 |
| | | | | | BCKDK | 0.00 | 2.18 | 1 | 1 |
| | | | | | ECHS1 | 0.00 | 7.24 | 1 | 1 |
| | | | | | GPX8 | 0.00 | 3.35 | 1 | 1 |
| | | | | | AGPAT1 | 0.00 | 2.83 | 1 | 1 |
| | | | | | PPAPDC2 | 0.00 | 3.39 | 1 | 1 |

**Table S1. Related to Figure2A - SF-25 target candidates identified by immuno-precipitation and mass spectrometry.** Target identification was conducted by a biochemical approach using immuno-precipitation and mass spectrometry analysis of pulled-down proteins from MDA-MB-468 tumor cell lysates. Two bands corresponding to 42kDa and 27kDa were sent for identification to Aulesa Biosciences. 138 proteins were identified in total using MASCOT.

# TableS2

| SF-25 binding score | | | | | | | | |
|---|---|---|---|---|---|---|---|---|
| Binding Panel 1<br>Takahashi *et al.* 1988 | cell line | A-427 | A-498 | AN3-CA | BT-20 | C-33 A | Calu-3 | Caov-3 |
| | binding score | 4000 | 15000 | 1000 | 500 | 1000 | 4000 | 7000 |
| | cell line | Hela | Hep 3B2.1-7 | Hep G2 | IGROV1 | JEG-3 | LS 180 | PLC/PRF/5 |
| | binding score | 20000 | 2000 | 12500 | 2500 | 5000 | 10500 | 4000 |
| | cell line | SK-CO-1 | SK-HEP-1 | SK-LU-1 | SK-MEL-5 | SK-UT-1 | SW403 | WiDr |
| | binding score | 3000 | 2500 | 2600 | 8000 | 4000 | 5000 | 4700 |
| Binding Panel 2<br>IgE | cell line | A2058 | A-375 | CAL51 | Capan-1 | HCC1954 | IGROV1 | MDA-MB-231 |
| | binding score | 11023 | 4331 | 3226 | 1861 | 7241 | 3226 | 1108 |
| | cell line | MDA-MB-468 | MIA-Paca-2 | PANC-1 | SK-BR-3 | SUM 149PT | WM983B | |
| | binding score | 1587 | 2419 | 70 | 8312 | 4891 | 6924 | |
| Binding Panel 3<br>IgG1 | cell line | A2058 | HeLa | LS 180 | IGROV1 | MDA-MB-231 | MDA-MB-468 | PaTu 8988t |
| | binding score | 33551 | 27071 | 12122 | 11297 | 6232 | 9952 | 17442 |

**Table S2. Related to Figure2A - Binding panels for bioinformatical target identification.**
Relative binding scores generated for three independent binding screens with the SF-25 antibodies. Binding scores for Binding Panel 1 were generated from a radioligand binding assay with the SF-25 murine IgG1 antibody in (Takahashi et al., 1988). Binding scores for Binding Panel 2 were generated from flow cytometry binding assay with the SF-25 chimeric IgE antibody [Figure1C]. Binding scores for Binding Panel 3 were generated from flow cytometry binding assay with the SF-25 chimeric IgG1 antibody [Figure1C].

## Figure S1

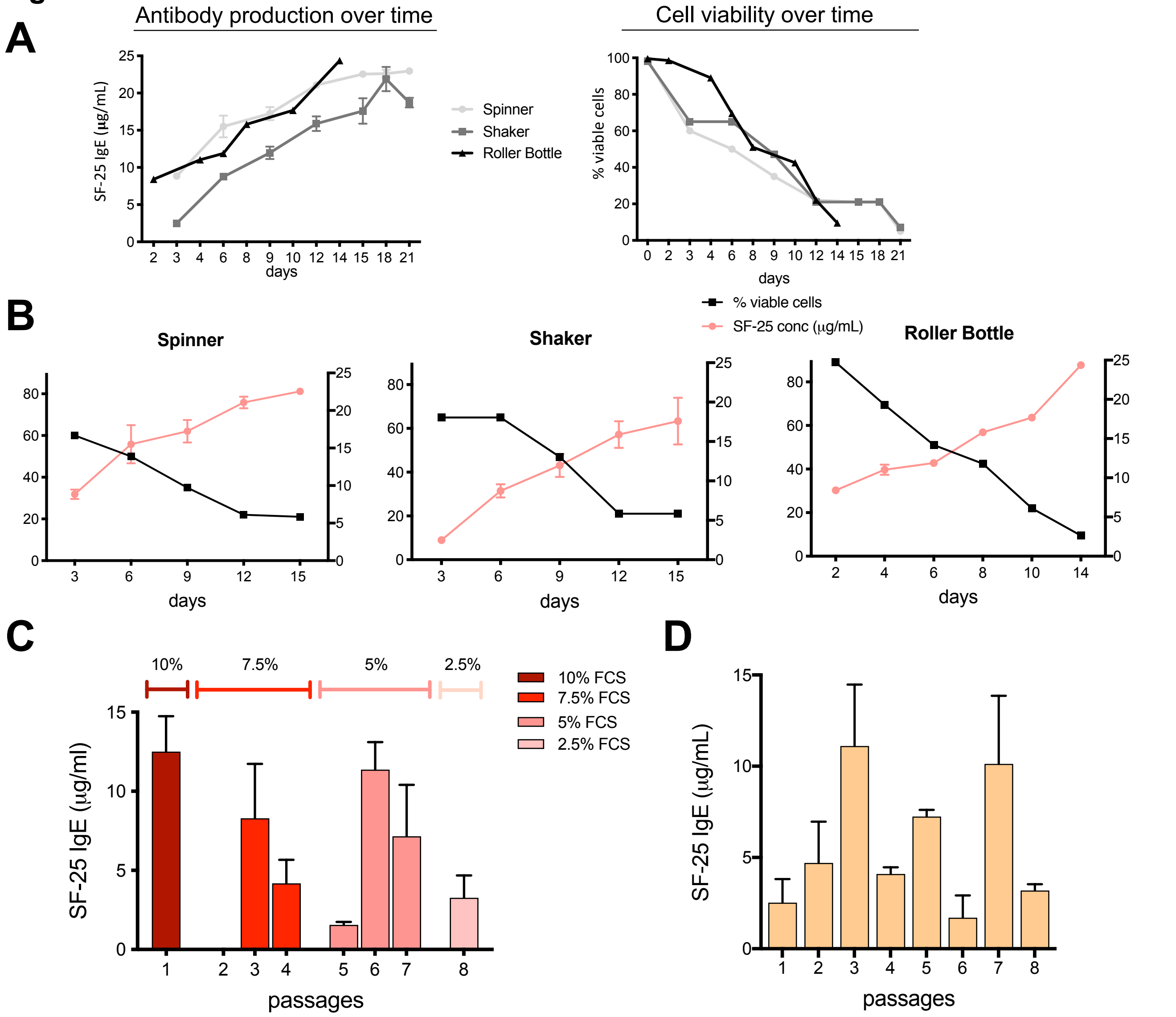


**Figure S1. Related to Figure 1A&B - Evaluation of multiple cell culture vessels and media for SF-25 IgE antibody production.**
(A) Different cell culture vessels were evaluated, and measurements were taken of both the SF-25 IgE concentration in the supernatant and of the cell viability over time. The graph on the left reports the yield of SF-25 IgE antibody over time across different culture vessels, while the graph on the right depicts the % of viable cells over the time in the same vessel types. (B) SF-25 IgE antibody concentration values and cell viability values were analysed for each cell culture vessel individually. Cells were seeded in each vessel with the same density of $5x10^5$ cells/ml and they were added fresh medium to the maximum working volume after three days. From day 3 onwards cells were not fed and left to starve until cell culture was harvested. (C) Sp2/0 cells can be adapted to serum-reduced condition and maintain SF-25 IgE production and secretion in the supernatant. Error bars represent SEM. (D) Cell viability was investigated over time and after every cell passage in ADCF cell culture, showing how cells keep a higher vitality rate even in serum optimised conditions. Error bars represent SEM.

## Figure S2

```
        10         20         30         40         50
MELQPPEASI AVVSIPRQLP GSHSEAGVQG LSAGDDSELG SHCVAQTGLE
        60         70         80         90        100
LLASGDPLPS ASQNAEMIET GSDCVTQAGL QLLASSDPPA LASKNAEVTG
       110        120        130        140        150
TMSQDTEVDM KEVELNELEP EKQPMNAASG AAMSLAGAEK NGLVKIKVAE
       160        170        180        190        200
DEAEAAAAAK FTGLSKEELL KVAGSPGWVR TRWALLLLFW LGWLGMLAGA
       210        220        230        240    *  **250
VVIIVRAPRC RELPAQKWWH TGALYRIGDL QAFQGHGAGN LAGLKGRLDY
       260        270        280        290        300
LSSLKVKGLV LGPIHKNQKD DVAQTDLLQI DPNFGSKEDF DSLLQSAKKK
       310        320        330        340        350
SIRVILDLTP NYRGENSWFS TQVDTVATKV KDALEFWLQA GVDGFQVRDI
       360        370        380        390        400
ENLKDASSFL AEWQNITKGF SEDRLLIAGT NSSDLQQILS LLESNKDLLL
       410        420        430        440        450
TSSYLSDSGS TGEHTKSLVT QYLNATGNRW CSWSLSQARL LTSFLPAQLL
       460        470        480        490        500
RLYQLMLFTL PGTPVFSYGD EIGLDAAALP GQPMEAPVML WDESSFPDIP
       510        520        530        540        550
GAVSANMTVK GQSEDPGSLL SLFRRLSDQR SKERSLLHGD FHAFSAGPGL
       560        570        580        590        600
FSYIRHWDQN ERFLVVLNFG DVGLSAGLQA SDLPASASLP AKADLLLSTQ
   ***  610    ****   620        630
PGREEGSPLE LERLKLEPHE GLLLRFPYAA
```

**Figure S2. Related to Figure 2B - Amino acid sequence of SLC3A2 – identified peptides by immuno-mass spectrometry.**

Peptides of the identified SLC3A2 protein are highlighted in the amino acid sequence. The sequence was obtained from UniProt (accession number=P08195).

# Figure S3

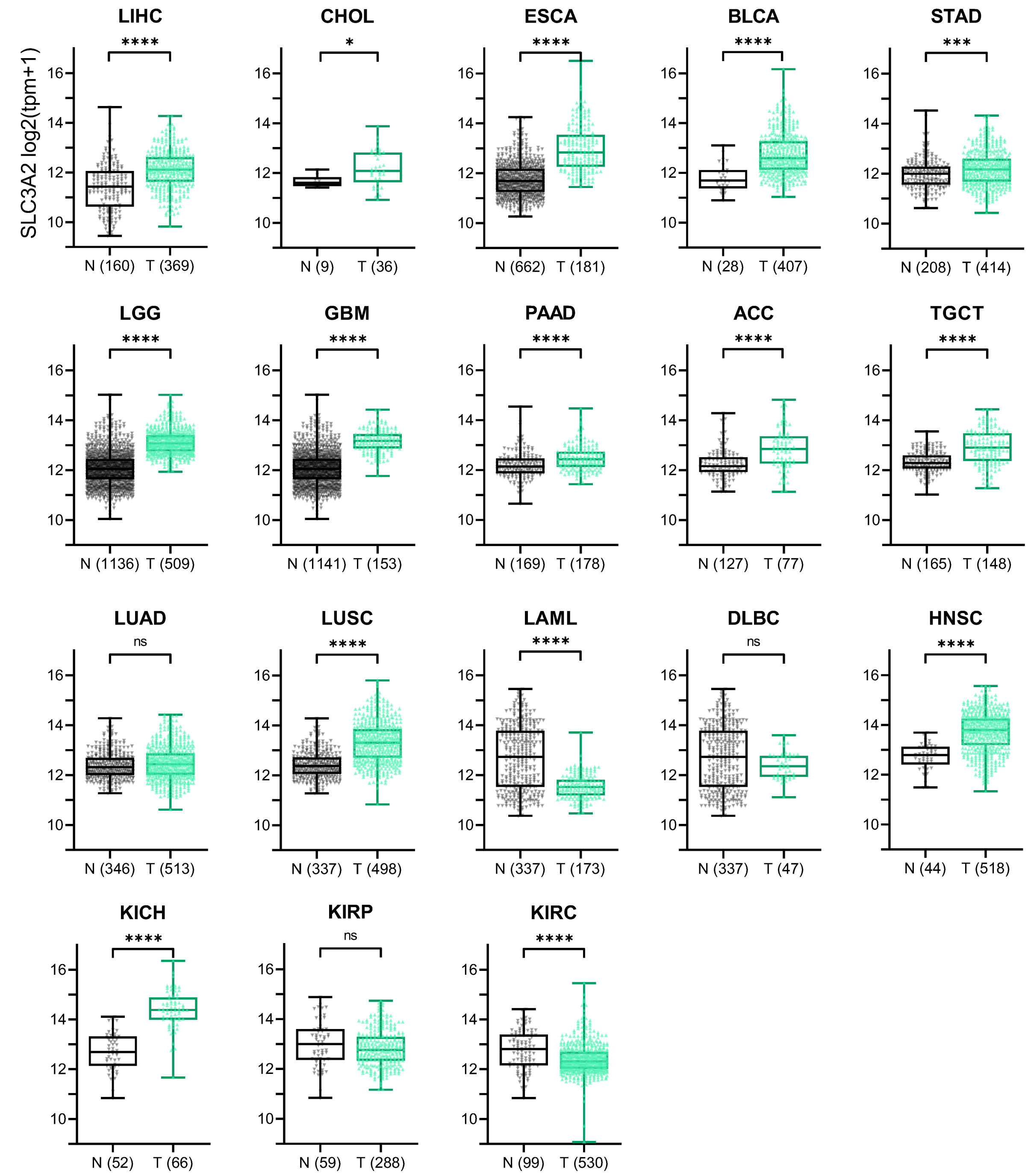


**Figure S3. Related to Figure 3 - *SLC3A2* gene expression is enhanced in different malignancies compared with equivalent normal tissues.**

Expression of *SLC3A2* in normal (N) versus tumor (T) tissues of different origins. Tumor types are described in Supplementary Materials and Methods - *SLC3A2* differential expression study. Mann-Whitney t-test was performed; ns = non-significant; * = p-value<0.05; ** = p<0.01; *** = p<0.005; **** = p<0.001.

## Figure S4

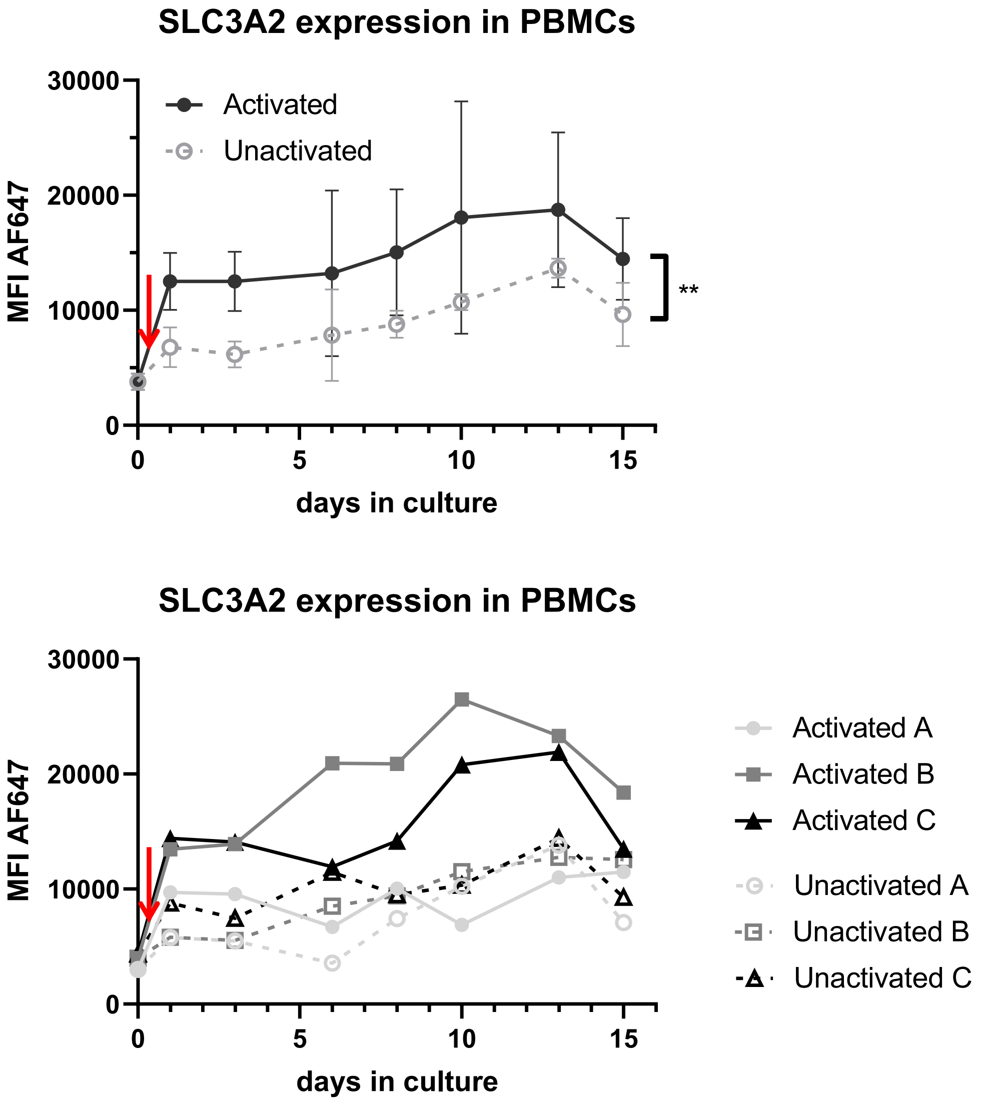


**Figure S4. SLC3A2 expression in PBMCs *in vitro*.**

Grouped (top) and individual curves (bottom panel) of SLC3A2 expression in activated (plain) and unactivated (empty symbols) PBMCs cultured 15days *in vitro* (n=3 individual donors). Activation is represented by an arrow. Mann-Whitney two-tailed analysis was performed excluding values before activation; **p=0.0041.

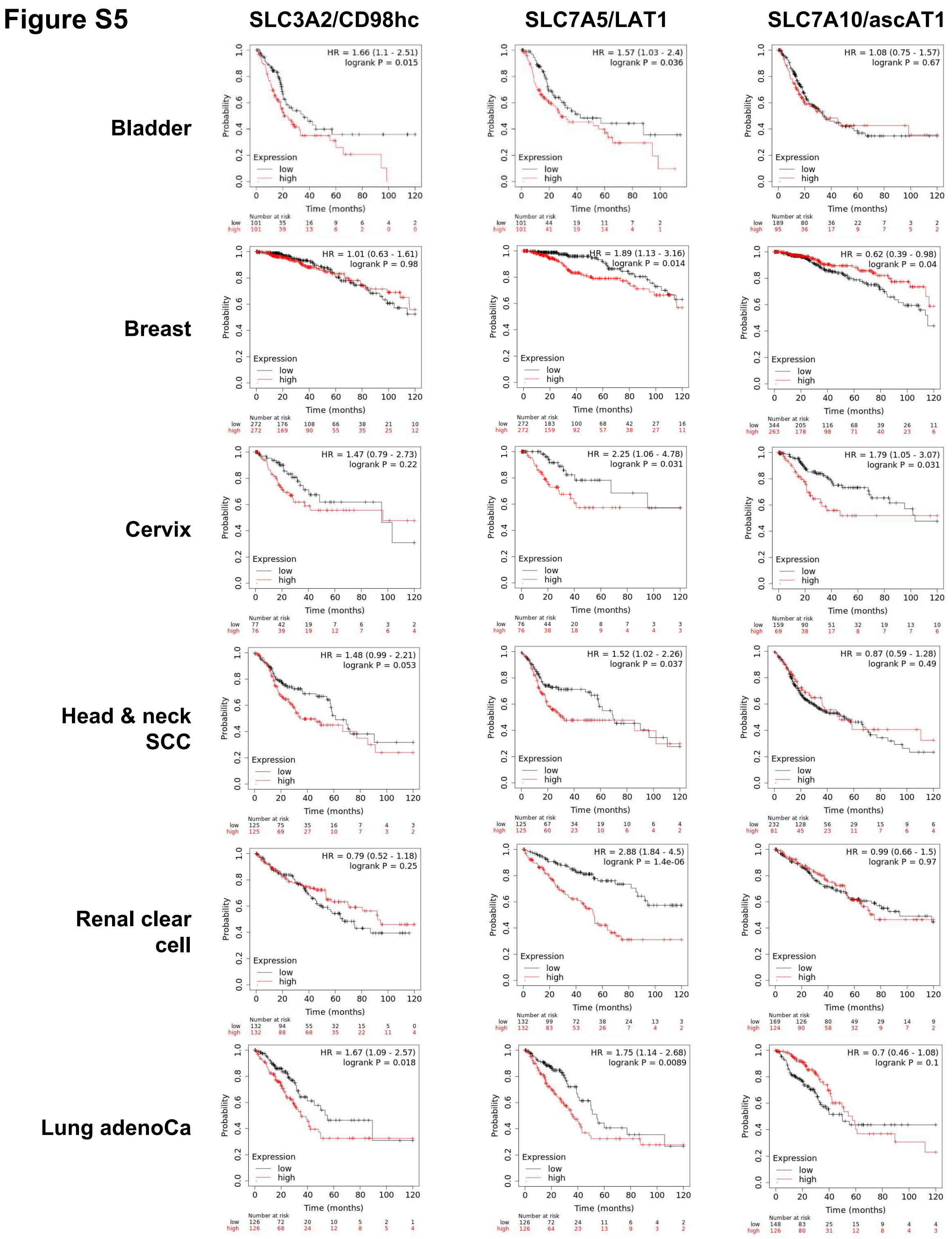


**Figure S5. Related to Figure 3 - Survival by expression of *SLC3A2*, *SLC7A5* and *SLC7A10* in human cancers.**
Kaplan-Meier curves of survival over 10 years for patients with bladder, breast, cervical, head and neck squamous cell carcinoma, renal clear cell carcinoma and lung adenocarcinoma by *SLC3A2*, *SLC7A5* and *SLC7A10* expression. High (red) and low (black) expression at baseline**.**

## Figure S6

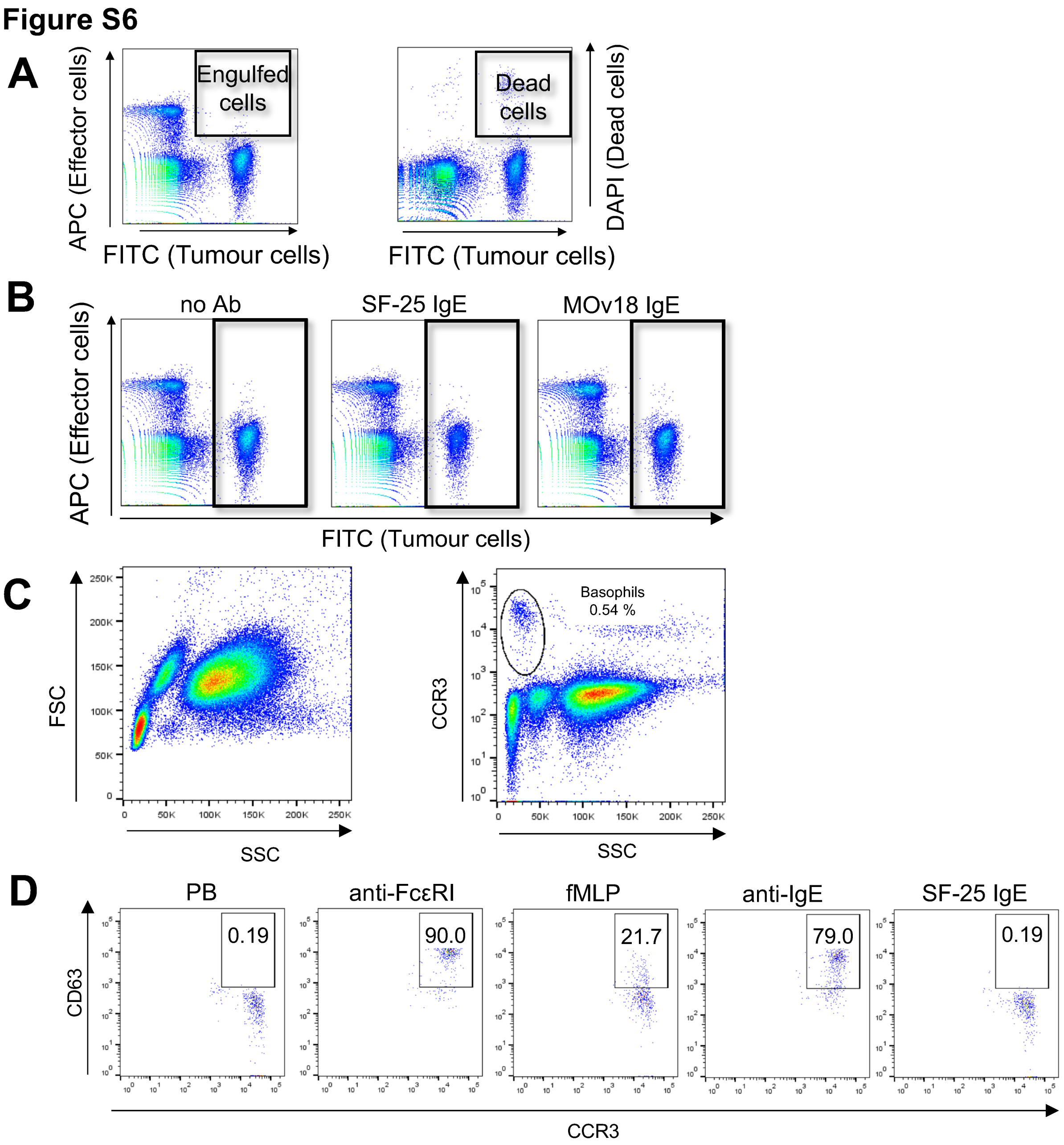


**Figure S6. Related to Figure 5(C-D)- Representative dot plots of ADCC/ADCP *in vitro* and of the Basophil Activation Test *ex vivo***

(A) Representative flow cytometry dot plots depicting the gating strategy to visualise the population of engulfed cells (left) and the population of dead target cells (right). (B) Representative flow cytometry plots depicting cell populations of effector (anti-CD89-APC) and target (FITC+) cells during ADCC/ADCP assay. The loss of tumour cells can be appreciated in the sample treated with SF-25 IgE when compared to controls. (C) Flow cytometric gating strategy to analyze the basophil population in unfractionated human blood samples. Together with analysis of the physical parameters two basophil-identifying markers were included in the flow cytometry analysis: CD63, an activation marker and CCR3 a basophil cell subset-specific surface molecule. (D) Representative flow cytometry plots showing the gating strategy to detect basophil activation (conditions tested included PB=patient baseline, anti-FcεRI, fMLP, anti-IgE).

# Figure S7

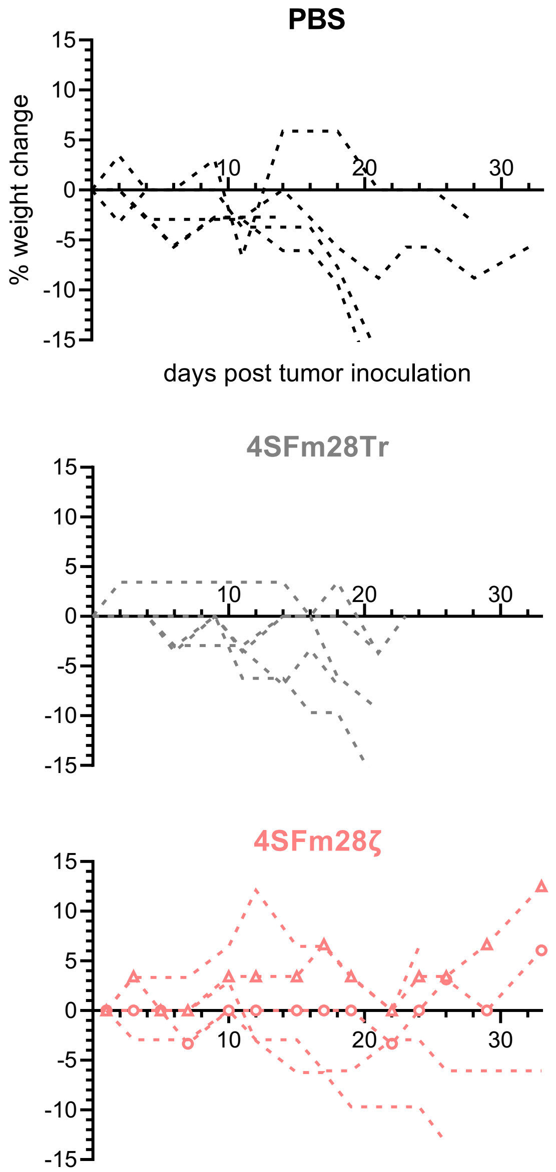


**Figure S7. Related to Figure 7G - SF-25 based CAR T cell *in vivo* toxicity.** Individual weight curves for mice in PBS treated (top), truncated CAR T cells (middle) and SF-25 second generation CAR T cells (bottom panel). The two long-term remission mice are highlighted with round and square symbols on the curve.
